# A modular method for the extraction of DNA and RNA, and the separation of DNA pools from diverse environmental sample types

**DOI:** 10.3389/fmicb.2015.00476

**Published:** 2015-05-19

**Authors:** Mark A. Lever, Andrea Torti, Philip Eickenbusch, Alexander B. Michaud, Tina Šantl-Temkiv, Bo Barker Jørgensen

**Affiliations:** ^1^Department of Bioscience, Center for Geomicrobiology, Aarhus UniversityAarhus, Denmark; ^2^Department of Chemistry, University of Duisburg-EssenEssen, Germany; ^3^Department of Land Resources and Environmental Sciences, Montana State UniversityBozeman, MT, USA; ^4^Microbiology Section, Department of Bioscience, Aarhus UniversityAarhus, Denmark; ^5^Department of Physics and Astronomy, Stellar Astrophysics Centre, Aarhus UniversityAarhus, Denmark

**Keywords:** DNA, RNA, extraction, environmental sample, low biomass, modular, intracellular, extracellular

## Abstract

A method for the extraction of nucleic acids from a wide range of environmental samples was developed. This method consists of several modules, which can be individually modified to maximize yields in extractions of DNA and RNA or separations of DNA pools. Modules were designed based on elaborate tests, in which permutations of all nucleic acid extraction steps were compared. The final modular protocol is suitable for extractions from igneous rock, air, water, and sediments. Sediments range from high-biomass, organic rich coastal samples to samples from the most oligotrophic region of the world's oceans and the deepest borehole ever studied by scientific ocean drilling. Extraction yields of DNA and RNA are higher than with widely used commercial kits, indicating an advantage to optimizing extraction procedures to match specific sample characteristics. The ability to separate soluble extracellular DNA pools without cell lysis from intracellular and particle-complexed DNA pools may enable new insights into the cycling and preservation of DNA in environmental samples in the future. A general protocol is outlined, along with recommendations for optimizing this general protocol for specific sample types and research goals.

## Introduction

The extraction of nucleic acids followed by downstream sequencing provides unparalleled insights into microbial community compositions in complex environmental samples. Since the advent of polymerase chain reaction (PCR) and sequencing technologies, a wide range of nucleic acid extraction methods have been developed, with different methods excelling on different sample types. These methods typically share the goal to lyse the entire microbial population within a sample and to subsequently recover and purify the nucleic acids of lysed cells, so they can be used for downstream molecular biological assays. Molecular biological methods e.g., PCR, realtime PCR, blotting, spectroscopy, enzymatic assays, or nucleic acid sequencing are then used to quantify and characterize microbial populations based on their DNA and RNA extracts.

Nucleic acid extraction protocols employ many different strategies. Yet, most methods share three aims: (1) the comprehensive lysis of cells and extraction of intracellular nucleic acids into aqueous solution, (2) the removal of non-nucleic acid organic and inorganic molecules from resultant aqueous extracts, and (3) the minimization of nucleic acid losses throughout this purification process. Cell lysis is achieved mechanically, e.g., by grinding, freeze-thawing or bead-beating (e.g., Tsai and Olson, 1991; MacGregor et al., 1997; Hurt et al., 2001), enzymatically, by incubations with enzymes that hydrolyze cell wall and cell membrane components (e.g., Holben et al., 1988; Smalla et al., 1993; Zhou et al., 1996), and/or chemically, by detergents which solubilize lipid membrane components or chaotropic agents which perforate cell membranes by denaturing trans-membrane proteins (e.g., Czomczynski and Sacchi, 1987; Pitcher et al., 1989; Moré et al., 1994; Zhou et al., 1996). The subsequent purification is done by washing with organic solutions and/or detergents, such as phenol, chloroform, or cetyl trimethylammonium bromide (Ogram et al., 1987; Stahl et al., 1988; Porebski et al., 1997), precipitation with ethanol, isopropanol, or polyethylene glycol (e.g., Stahl et al., 1988; Paithankar and Prasad, 1991; Zhou et al., 1996; Porteous et al., 1997; Wang et al., 2011), and/or filtration through silica-columns, magnetic beads, ion-exchange resins, or gels (Zhou et al., 1996; Hurt et al., 2001; Arbeli and Fuentes, 2007; Zhao et al., 2008; Tanaka et al., 2009). Moreover, because nucleic acids adsorb to positively charged surfaces, chemical carriers are added in many protocols, to coat charged surfaces prior to cell lysis. Chemical carriers often contain phosphate and include inorganic phosphate species, nucleic acid building blocks, and even nucleic acids (Rogers and Bendich, 1985; Holben et al., 1988; Pietramellara et al., 2001; Cai et al., 2006).

While nucleic acid extraction in the 1980s and 1990s mainly relied on handcrafted chemical solutions and extraction protocols (e.g., Czomczynski and Sacchi, 1987; Holben et al., 1988; Tsai and Olson, 1991; Zhou et al., 1996) there has been a shift toward increased use of commercial extraction kits since then (e.g., Dhillon et al., 2005; Francis et al., 2005; Rudi et al., 2005; Schippers and Neretin, 2006; Edgcomb et al., 2010; Borrel et al., 2012). These kits have many advantages, which include prepared reagents and streamlined extraction procedures. Several kits produce adequate nucleic acid yields across wide ranges of samples (e.g., Roose-Amsaleg et al., 2001; Webster et al., 2003; Inagaki et al., 2006; Amaral-Zettler et al., 2009; Coolen et al., 2011). Use of the same kit by different individuals facilitates cross-comparing molecular biological data sets due to method standardization. Despite these advantages, no universal extraction kit that performs best for all sample types and research goals has been developed (Martin-Laurent et al., 2001; Lombard et al., 2011). Part of the reason might be that environmental samples and microbes have divergent properties that make the design of universally optimized methods futile. Consequently, there may be a benefit to adjusting extraction protocols to meet specific sample characteristics and research requirements. The systematic fine-tuning of commercial extraction kits for applications with specific environmental sample types is, however, difficult because reagent recipes are typically proprietary.

Optimizations of extraction procedures, e.g., with respect to lysis efficiency and recovery, are especially critical in studies where nucleic acids are used to infer microbial population size or community structure (Martin-Laurent et al., 2001; Lipp et al., 2008; Morono et al., 2014). Similarly, adjustments to existing protocols are often essential with samples possessing low population densities, high adsorptive properties, or complex matrices, where the extraction of any amplifiable nucleic acids can present a challenge Sørensen et al., 2004; Alain et al., 2011; Nielsen et al., 2014). Moreover, the selection of commercial kits decreases dramatically when specific extraction requirements need to be met. An example is the wide selection of kits for DNA or RNA extraction and the comparatively much smaller selection of kits for extraction of both DNA and RNA. For unconventional applications that are only of interest to a small community of scientists, e.g., the separate extraction of different DNA pools (e.g., Ogram et al., 1987; Corinaldesi et al., 2005), commercial kits may not even exist. As a result, despite the added effort of preparing reagents and more time-intensive extraction protocols, handcrafted extraction methods remain relevant to certain fields of nucleic acid-based research (Martin-Laurent et al., 2001; Schneegurt et al., 2003; Lipp et al., 2008; Bey et al., 2010; Alain et al., 2011; Alawi et al., 2014; Morono et al., 2014).

Recognizing the possibility that a universally best extraction method may never exist, we design a modular nucleic acid extraction protocol that provides high nucleic acid yields across a diverse range of environmental sample types. Most samples tested are marine sediments, ranging from surface to deep subsurface, and eutrophic to ultraoligotrophic, however, we also include lacustrine sediment, igneous rock, water, and air samples. With only minor modification, this method can be adapted to meet specific research needs, such as the simultaneous extraction of DNA and RNA, and the separate extraction of two different DNA pools. The two DNA pools are an aqueous-extractable “soluble DNA” (sDNA) pool, that is likely dissolved or adsorbed to the initial sediment matrix, and a “non-soluble” (nsDNA) pool, that is within cells or particle-complexed in the initial sediment matrix, and requires cell lysis and chaotropic treatment to become water soluble. In the past, these two DNA pools have been termed “extracellular DNA” and “intracellular DNA,” respectively (Ogram et al., 1987; Corinaldesi et al., 2005; Alawi et al., 2014; for more details see “Discussion”).

To develop this modular nucleic acid extraction method, we use an iterative approach. High-biomass, organic-rich coastal sediment is used as initial test material. Starting with a suboptimal original protocol, we incrementally improve DNA yields by modifying the original protocol through trials of single-variable permutations. Using the best-performing permutation as the reference method in each new trial, we continue our trials until DNA yields no longer increase. Subsequently, we test the best DNA extraction protocol for coastal sediment on other sample types, including buried soil (ultra)oligotrophic freshwater and marine sediments, deeply buried and ancient subseafloor sediments, subseafloor basalt, lake water samples, and air samples. With a subset of these additional sample types we perform further tests to improve DNA yields on these particular samples. We then test modifications that enable the separation of sDNA and nsDNA pools, the simultaneous extraction of DNA and RNA, and conclude with comparing DNA and RNA yields to commercial kits by MO BIO Laboratories and MP Biomedicals.

## Methods

### Sampling

An overview of the samples tested is presented in Table 1.

**Table 1:** **Details of samples used to test this extraction method**.

**Location**	**Site/Station**	**Latitude**	**Longitude**	**Depth (mbsl)**	**Depth (mbsf/mblf)**	**Sample type**
Aarhus Bay (Kattegat)	M5	56.103°N	10.458°E	27	1.2	Clay-rich mud, MGZ
	MIMOSA	56.260°N	10.469°E	16	1.2	“ “ “, MGZ
	M1	56.118°N	10.347°E	15	0.05	“ “ “, BTZ
	M1	“	“	15	0.80	“ “ “, SRZ
	M1	“	“	15	1.60	“ “ “, SMTZ
	M1	“	“	15	3.10	“ “ “, MGZ
	M1	“	“	15	10.55	Terrestrial soil, MGZ
Namibian Shelf	GeoB12806	25.001°S	14.389°E	133	0.04	Sulfidic, mudbelt, SRZ
	GeoB12806	“	“	133	~2.8	“ “ “
Bering Sea	U1342B-1H-2	54.828°N	176.917°E	830	1.75	Silty clay, SRZ
	U1343E-80X-5	57.556°N	175.817°W	1968	712	Diatom clay, ash, MGZ
	U1344C-1H-3	59.050°N	179.203°W	3184	3.35	Diatom clay, SRZ
	U1344A-7H-2	59.050°N	179.203°W	3183	59	Silt/sand, MGZ
Guaymas Basin	Orange mat (M14)	27.008°N	111.407°W	~2000	0.02	Petroleum-rich, hydrothermal sediment, SRZ
	“ “	“	“	~2000	0.31	“ “ “ “
	Yellow mat (M14)	“	“	~2000	0.02	“ “ “ “
Peru Trench	ODP 1230A-21-3	9.113°S	80.584°W	5086	190	Diatom clay/ooze, MGZ
Off Shimokita Peninsula	10R-1	41.178°N	142.201°E	1180	1630	Silt/fine sand, MGZ
	24R-3	“	“	1180	1990	Medium sand, MGZ
	C0020 (165LMT)	“	“	1180	N/A	Drilling mud
	C0020 (61SMT)	“	“	1180	947	Unwashed drill cutting, silt
	C0020 (61SMT)	“	“	1180	947	Washed drill cutting, silt
Bornholm Basin, Baltic Sea	Station 024 7GC	55.250°N	15.436°E	94	~10	Ice lake clay, FeRZ, SRZ
Subglacial Lake Whillans	Drill Site	84.237°S	153.614°W	800^#^	0.05	Subglacial clay, oxic
South Atlantic Gyre	GeoB12815	27.237°S	10.000°E	4662	0.01	Pelagic red clay, oxic
South Pacific Gyre	SPG 1	23.850°S	165.650°W	5697	0.10	Pelagic red clay, oxic
	SPG 6	27.917°S	123.167°W	3738	0.01	“ “ “ “
	SPG 6	“	“	3738	1.12	“ “ “ “
	U1371F-1H-1	45.964°S	163.184°W	5301	1.1	Diatom clay/ooze, microoxic
Juan de Fuca Ridge Flank	1362A-17R-3	47.761°N	127.761°W	2672	462.1	Massive basalt, vein
Danish lake, north Jutland	N/A	57.360°N	9.941°E	Surface	N/A	Eutrophic water, oxic
Greenland glacial lake	N/A	65.312°N	50.202°W	Dried up	N/A	Glacial till, MGZ
Aarhus University	2nd floor balcony	56.166°N	10.200°E	N/A	N/A	Outdoor urban air

*All samples were stored frozen prior to extraction except samples from Aarhus Bay Stations M5 and MIMOSA, which were stored in a gas-tight plastic bag at 4°C. “Orange mat” and “Yellow mat” refer to sites that were covered by orange- and yellow-colored Beggiatoa mats, M14 refers to “Marker 14.” Mbsf, meters below seafloor; mblf, meters below lakefloor; N/A, not applicable; BTZ, bioturbation zone; SRZ, sulfate reduction zone; FeRZ, iron reduction zone; MGZ, methanogenesis zone*.

# *, meters below ice surface*.

#### Sediment samples

For initial tests, we used organic-rich coastal sediment from Aarhus Bay stations M5 and MIMOSA. Whole round gravity cores were transferred to gas-tight bags to minimize oxygen exposure and stored at 4°C. All other marine sediment samples and the Greenland glacial lake sample came from minimally contaminated inner parts of sediment cores and were stored at −80°C. Prior to sampling, these sediment samples were allowed to warm without fully thawing, i.e., only until sediments were soft enough for subsampling with a sterile metal spatula. Samples collected from Subglacial Lake Whillans were sampled as described previously (e.g., Priscu et al., 2013). Subsamples were weighed and kept on ice until the start of extractions. In tests comparing nucleic acid extraction efficiencies of different extraction treatments, samples were homogenized prior to weighing to reduce variability in DNA content between sample aliquots.

#### Drill cutting and drilling mud samples

Samples of drill cuttings and drilling mud were obtained during riser drilling aboard the R/V Chikyu during Integrated Ocean Drilling Program (IODP) Expedition 337 in 2012. These samples were frozen at −80°C immediately after arrival on deck.

#### Rock sample

The basalt core was kept at −80°C until sampling. The exterior of basal cores was decontaminated by washing and flaming as outlined in Lever et al. (2006). Sampling of core interiors was done as described in Lever et al. (2013).

#### Air samples

Air samples of ~500 m^3^ volume were collected with an impinger sampler from a 2nd floor balcony. The impinger sampler streamed air through 2 L of “high-salt sampling solution,” in which microbial cells are captured (Šantl-Temkiv et al., 2013). The high-salt sampling solution, consisting of 25 mM sodium citrate, 10 mM EDTA, and ammonium sulfate (450 g L^−1^, pH 5.2), was designed to preserve RNA throughout sample collection. Captured cells were subsequently concentrated onto Sterivex™ filter units (0.22 μm, Millipore), which had been capped with Luer-Lok caps. Further details in Supplementary Online Material (SOM).

#### Water samples

Water samples were filtered through 11-μm filters to remove algal cells. Microbial cells from half a liter of sample were concentrated onto Sterivex™ filter units. RNA was fixed with 2 mL of high-salt solution (see “Air samples”) and capped with inlet and outlet Luer-Lok caps. Filters were kept at 4°C for 3 h. Afterward the high salt solution was washed off with 10 mL of sterile deionized water and the filters were frozen at –80°C. Further details in SOM.

### Extraction tests

We tested mechanical, chemical and enzymatic cell lysis methods, methods to prevent nucleic acid adsorption, nucleic acid purification methods, precipitation assays, and commercial kits. An overview of all variables and kits tested is shown in Table 2.

**Table 2:** **Overview of variables examined in nucleic acid extraction tests**.

**(A) MECHANICAL LYSIS**		
**Method**	**Purpose**	**Approach**
Bead-beating	Break up particles containing cells, dislodge cells, and/or mechanically destroy cells	Shaken for 1 min on FastPrep FP120 Homogenizer (Thermo Savant) or TissueLyser LT 25000 (Qiagen), or for 10 min on Vortex Genie at maximum setting (3000) with 0.1-mm zirconia/silica beads (Biospec Products) added to ~15% of 2-mL screw cap tube volume
Homogenizer	Cell dislodging and cracking	Pro Scientific 300D (Pro Scientific); homogenized for 2 min at 1000, 2000, 3000, 4000, 6000, 8000, 10,000, or 15,000 rpm.
Freeze-thawing	Cell cracking by ice crystals	Deeply frozen at -80°C
Heat	Heat-stimulation of chaotropic chemicals, surfactants, protein, lipid-, and peptidoglycan-degrading enzymes	Gently mixed at 50°C for 1-h intervals on thermomixers (Eppendorf) set to 600 rpm, or in shaker incubators
**(B) CHEMICAL/ENZYMATIC LYSIS**		
**Chemical**	**Purpose**	**Treatment**
Tris Hydrochloride (Tris-HCl)	Buffers pH of lysis solutions at levels that are suited for enzymatic treatments	Tested 10–300 mM
Na_2_EDTA (EDTA)	Inactivates nucleases	Tested 10–100 mM
Guanidium hydrochloride	Denatures proteins	800 mM; with and without 50°C incubation
Triton X-100	Disrupts cell membranes	0–2% vol/vol; with and without 50°C incubation
Sodium dodecyl sulfate (SDS)	Anionic surfactant that disrupts cell membranes and denatures proteins	0–4% vol/vol using 20% SDS stock solution, with and without 50°C incubation
Phenol-chloroform-isoamylalcohol (PCI; 25:24:1)	Phenol denatures proteins. Chloroform dissolves/binds nonpolar constituents. Isoamylalcohol stabilizes interface of phenol-choroform and aqueous extract	Compared treatments with PCI to ones without PCI during initial lysis
Cetyl trimethylammonium bromide (CTAB)	Cationic surfactant that disrupts cell membranes	Concentration range: 0–2%; 1-h incubation at 50°C followed by 1-h incubation at 65°C
Proteinase K	Destroys proteins (structural, membrane-bound and enzymatic)	Tested 0–4 μg mL^−1^
Lysozyme (muramidase)	Hydrolyzes N-acetylmuramic acid N-acetylglucosamine bonds	Tested 0–0.5 μg mL^−1^
Lipase Typ7	Hydrolysis of lipids	Concentration of 0–0.5 μg mL^−1^
2-hydroxyquinoline	Antioxidant; prevents phenol oxidation.	Tested 0–0.1% wt/vol in PCI
β-mercaptoethanol	Antioxidant, prevents phenol oxidation, and reduces disulfide bonds	Added 0–0.2% vol/vol to lysis buffer I or PCI
tris(2-carboxyethyl)phosphine (TCEP)	Same as β-mercaptoethanol	Supplied to lysis buffer I at 0–10 mM
**(C) ADSORPTION PREVENTION**		
**Chemical**	**Purpose**	**Treatment**
pH		pH 5–10
Sodium pyrophosphate (pyroPO_4_; P_2_O^4-^_7_)	Bind competitively with nucleic acids onto charged mineral surfaces.	Dose g^−1^ sample: 0–0.045 g or 0–400 μmol PO_4_
Sodium hexametaphosphate (hexaPO_4_; (PO_3_)^6-^_6_)		Dose g^−1^ sample: 0-0.061 g or 0–600 μmol PO_4_
Deoxynucleoside triphosphates (dNTPs)		Dose g^−1^ sample: 0-0.029 g or 0–180 μmol PO_4_
30-base pair PCR product		Dose g^−1^ sample: 3.3 × 10^−7^g or 0.001 μmol PO_4_ g^−1^
Salmon sperm DNA		Dose g^−1^ sample: 0-0.005 g or 0–15 μmol PO_4_
**(D) PURIFICATION**		
**Chemical**	**Purpose**	**Treatment**
CTAB	Removal of polysaccharides	0–2%*
Polyvinylpolypyrrolidone (PVPP)	Removal of polyphenolic compounds (e.g. fulvic and humic acids)	0–0.2%
Phenol (pH 7.9)	Denatures proteins. Removes proteins, lipids and detergents by dissolution or accumulation at aqueous interface.	1:1 (v/v) extract, followed by PCI and chloroform wash
Phenol-chloroform-isoamylalcohol (PCI; 25:24:1; pH 7.9)	Removes proteins, lipids and detergents by dissolution or accumulation at aqueous interface.	1:1 (v/v) extract and PCI, followed by 1–2 chloroform washes
Chloroform-isoamylalcohol (CI; 24:1)	Removes residual phenol, proteins, lipids and detergents by dissolution or accumulation at aqueous interface	1:1 (v/v) extract and CI, 1–3 washes
**(E) PRECIPITATION**		
**Chemical(s)**	**Purpose**	**Treatment**
Ethanol-NaCl	DNA concentration	Add NaCl to 1.2–1.8 M, then add 2.5 V ethanol
Isopropanol-NaCl	DNA and RNA concentration	Add NaCl to ~0.8 M, then add 1.5 V isopropanol
Isopropanol-Ammonium acetate	DNA and RNA concentration	Add NH^+^_4_-acetate to ~3.8 M, then add 1.5 V isopropanol
PEG 6000-NaCl	DNA concentration	Add 2 V solution (30% PEG, 1.6 M NaCl)
PEG 8000-NaCl	DNA concentration	Add 2 V solution (30% PEG, 0.4–2 M NaCl)
PEG 8000-Ethanol-NaCl	DNA concentration	Add 2 V solution (75% ethanol with 1 M NaCl mixed with 4% *w/v* PEG 8000)
PEG 6000-NaAcetate/Acetic Acid	DNA concentration	0.1 V of 3 M Na acetate, 0.1 V of 1:1/9:1/99:1 (v:v) 3 M acetic acid + Na acetate, 0.1 V of 3 M acetic acid
PEG 6000-NaCl-MgCl_2_	Enhance DNA yield	0.1 V of 300 mM MgCl_2_ + 2 V (30% PEG, 1.6 M NaCl)
Precipitation temperature	Enhance DNA yield and purity	−20°C, 4°C, room temperature in dark
Centrifugal force	Enhance DNA yield	14,000×g, 20,000×g
**(F) COMMERCIAL PURIFICATION AND EXTRACTION KITS**	
**Name**		**Purpose**
CleanAll RNA/DNA Clean-up and Concentration Kit (Norgen Biotek) PowerClean DNA Clean-Up Kit (MO BIO Laboratories		Purify nucleic acid extracts after precipitation (Norgen: DNA and RNA, MO BIO: DNA only) for downstream enzymatic assays or PCR applications
PowerSoil DNA Isolation Kit (MO BIO Laboratories) PowerLyzer PowerSoil DNA Isolation Kit (MO BIO Laboratories) FastDNA SPIN Kit for Soil DNA Extraction (MP Biomedicals		Extraction and purification of DNA from soil and sediment for downstream enzymatic assays or PCR
PowerWater® Sterivex^TM^ DNA Isolation Kit (MO BIO Laboratories)		Extraction and purification of DNA from water samples for downstream enzymatic assays or PCR
RNA PowerSoil® Total RNA Isolation Kit (MO BIO Laboratories)		Extraction and purification of RNA from soil and sediment for downstream enzymatic assays or PCR

*The extraction components that were tested are shown in six subdivisions: (A) mechanical lysis (B) chemical/ enzymatic lysis (C) adsorption prevention (D) purification (E) precipitation, and (F) commercial purification and extraction kits. Chemicals that serve multiple functions are listed in more than one subdivision*.

#### Physical/mechanical lysis (Table 2A)

We investigated effects of bead-beating, use of a homogenizer, freeze-thawing, and heat treatment on nucleic acid yields.

#### Chemical/enzymatic lysis (Table 2B)

We compared effects of detergents (SDS, Triton X-100, and CTAB), enzymes (proteinase K, lipase, lysozymes), humic substance complexation agents (CTAB, PVPP), inclusion of PCI and reductants (2-hydroxyquinoline, β-mercaptoethanol, TCEP) and extraction/lysis buffer chemical composition (pH, Tris-HCl, EDTA, phosphate, guanidium hydrochloride, and sodium chloride).

#### Adsorption prevention (Table 2C)

We tested the effect of pH (range: 5–10), and adding variable doses of sodium pyrophosphate, sodium hexametaphosphate, dNTP, and salmon sperm DNA on DNA recovery.

#### Purification (Table 2D)

DNA purification methods involving chloroform, phenol (pH 7.9), chloroform-isoamylalcohol, and phenol-chloroform-isoamylalcohol (pH 7.9) were tested. We also tested adding the reductants 2-hydroxyquinoline, β-mercaptoethanol, and TCEP to prevent phenol oxidation.

#### Precipitation (Table 2E)

We compared nucleic acid precipitation methods involving ethanol-NaCl, isopropanol-ammonium acetate, isopropanol-NaCl, PEG 6000-NaCl, PEG 8000-NaCl, and PEG 8000-ethanol-NaCl at temperatures ranging from room temperature to −20°C. Further variables tested were adding different NaCl doses during ethanol-NaCl precipitation, adding MgCl_2_ or solutions containing sodium acetate, sodium acetate-glacial acetic acid, or glacial acetic acid during PEG 6000-NaCl precipitations, as well as the use of two different centrifugal forces to pellet nucleic acids after precipitation with PEG 6000-NaCl.

#### Commercial purification and extraction kits (Table 2F)

For post-extraction purification, we tested two purification kits. We also compared DNA and RNA yields obtained with our extraction protocol to those obtained with five commercial extraction kits.

#### Separation of DNA pools

We developed a protocol for the separate extraction of sDNA and nsDNA, with the aim to maximize DNA yields of both pools, while preventing cell lysis and false incorporation of nsDNA during the sDNA extraction process. To design this method, we examined the effects of pH, amount of PO_4_ added g^−1^ sediment, and sDNA extraction solution composition on the yields of sDNA and nsDNA (variables tested are included in Table 2D). We checked for cell lysis due to the sDNA extraction procedure by epifluorescence microscopic enumeration (Methodological details in SOM). To assess how other sDNA extraction methods compared to ours, we performed cell counts on sediments from which sDNA had been extracted using protocols by Ogram et al. (1987) and Corinaldesi et al. (2005). Finally, we examined size distributions of sDNA to gain insights into the cycling of sDNA in sediments.

### DNAse treatment and reverse transcription

To remove DNA, 10 μL of 10× Reaction Buffer and 2 μL TURBO™ DNase (Ambion) were added per 100 μL of extract and incubated on a Thermomixer Comfort (Eppendorf) shaking at 600 rpm for 30 min at 37°C. RNA was reverse-transcribed into cDNA using the Omniscript RT Kit (Qiagen) with random hexamer primers (Biomers.net) and ANTI-RNase (Ambion) as RNA inhibitor.

### Nucleic acid quantification

Nucleic acid extraction efficiency was assessed by fluorescence spectroscopic measurements and quantitative real-time PCR (qPCR) assays.

#### Quantification by fluorescence spectroscopy

DNA and RNA were quantified using a Nanodrop-3300 fluorospectrometer (Thermo Scientific). Standard curves were prepared using dilutions of lambda DNA/HindIII ladder (Invitrogen) for DNA and dilutions of 0.1–2 Kb RNA Ladder (Ambion) for RNA. Standard concentrations spanned 1 pg to 10 ng μL^−1^. DNA and RNA were stained with the fluorescent dyes Quant-iT™ PicoGreen and Quant-iT™ RiboGreen, respectively, using 25-fold dilutions of the manufacturer's dye solutions (both Invitrogen). All standards and dye solutions were prepared with the same buffers used for DNA or RNA dissolution/elution, due to the strong influence of buffer composition on fluorescence values. Duplicate or triplicate measurements on 2-μL aliquots were performed on final mixtures consisting of 3 μL of dye solution and 3 μL of standard or sample. These mixtures were thoroughly homogenized by vortexing, then centrifuged for 1 min at 10,000×g, and measured within 10 min of being homogenized, as quick processing was essential to measurement reproducibility. DNA or RNA solutions were kept on ice until addition of dye, then maintained at room temperature until measurement.

#### Quantification by qPCR

Bacterial and archaeal 16S rRNA gene copy numbers were quantified on a Roche Light Cycler 480. The primer combinations 8Fmod-338Rabc and Bac908F_mod-Bac1075R were used for Bacteria, whereas the primer combinations 806F-958R and 915Fmod-1059R were used for Archaea (Table 3). Standard curves were based on pGEM-T plasmids (Promega) with bacterial or archaeal 16S rRNA gene inserts. Each run included extraction and PCR negative controls. PCR inhibition was checked using 1:10 and 1:100 dilutions of extracts. All standards, controls, samples, and sample dilutions were run in duplicate or triplicate. Each 20-μL reaction mixture was composed of 10 μL Roche Light cycler master mix containing SYBR-Green, 1 μL of each 50 μM primer solution, 2 μL of 1 mg mL^−1^ bovine serum albumin, 2 μL DNA/copy DNA (cDNA) template solution, and 4 μL molecular-grade H_2_O. The qPCR protocol consisted of (1) 95°C polymerase activation for 5 min, followed by (2) 45-50 PCR cycles consisting of (a) denaturation for 30 s at 95°C (b) annealing for 30 s (temperatures in Table 3) (c) elongation for 30 s at 72°C, and (d) fluorescence measurement after 5 s at 80°C. Each qPCR run was concluded with (3) a stepwise melting curve from 95 to 55°C for 1 min, which was used to check for primer specificity.

**Table 3:** **16S rRNA gene primers used for qPCR examinations of bacterial and archaeal nucleic acid extraction efficiency**.

**Primer**	**Target**	**T*_m_* (°C)**	**Sequence (5′–3′)**	**References**
8Fmod	Bacteria	60	AGA GTT TGA TYM TGG CTC AG	Juretschko et al., 1998
338Rabc	Bacteria	60	ACW CCT ACG GGW GGC WGC	Daims et al., 1999
Bac908F_mod	Bacteria	60	AAC TCA AAK GAA TTG ACG GG	This study, modified from Ohkuma and Kudo (1998)
Bac1075R	Bacteria	60	CAC GAG CTG ACG ACA RCC	Ohkuma and Kudo, 1998
806F	Archaea	55	ATT AGA TAC CCS BGT AGT CC	Takai and Horikoshi, 2000
915Fmod	Archaea	55	AAT TGG CGG GGG AGC AC	Cadillo-Quiroz et al., 2006
958R	Archaea	55	YCC GGC GTT GAM TCC AAT T	DeLong, 1992
1059R	Archaea	55	GCC ATG CAC CWC CTC T	Yu et al., 2005

### Statistical analyses

Using the online statistical software program Wessa.net (www.wessa.net), we performed unpaired student *t*-tests to check whether differences in DNA yields between treatments and treatment permutations were statistically significant. This test was chosen after confirming—using the D'Agostino skewness test, Anscombe-Glynn kurtosis test, and Jarque-Bera Normality Test on a subset of data—that DNA yields within treatments and treatment permutations did not deviate significantly from normality. *P*-values below 0.05 were considered statistically significant.

## Results

We present the tests, which were instrumental to the design of this modular protocol for nucleic acid extraction. During the protocol development, progress in extraction yields was not always linear, with outcomes of downstream treatments resulting in modifications to upstream treatments late in the development. For the sake of clarity, we have organized the test results in the chronological order of the extraction protocol. After showing the DNA extraction test results, we present results for the extraction of RNA and the separate extraction of sDNA and nsDNA. We then compare DNA/RNA yields to ones obtained with widely used commercial kits and provide an overview of samples to which this protocol has been successfully applied.

### General protocol tests

#### Physical/mechanical lysis

We here only discuss effects of bead-beating and homogenizer treatment. Effects of freeze-thawing and heat treatments are discussed in the context of chemical/enzymatic lysis treatments, with which both were typically combined.

#### Bead-beating

**Figure 1:**
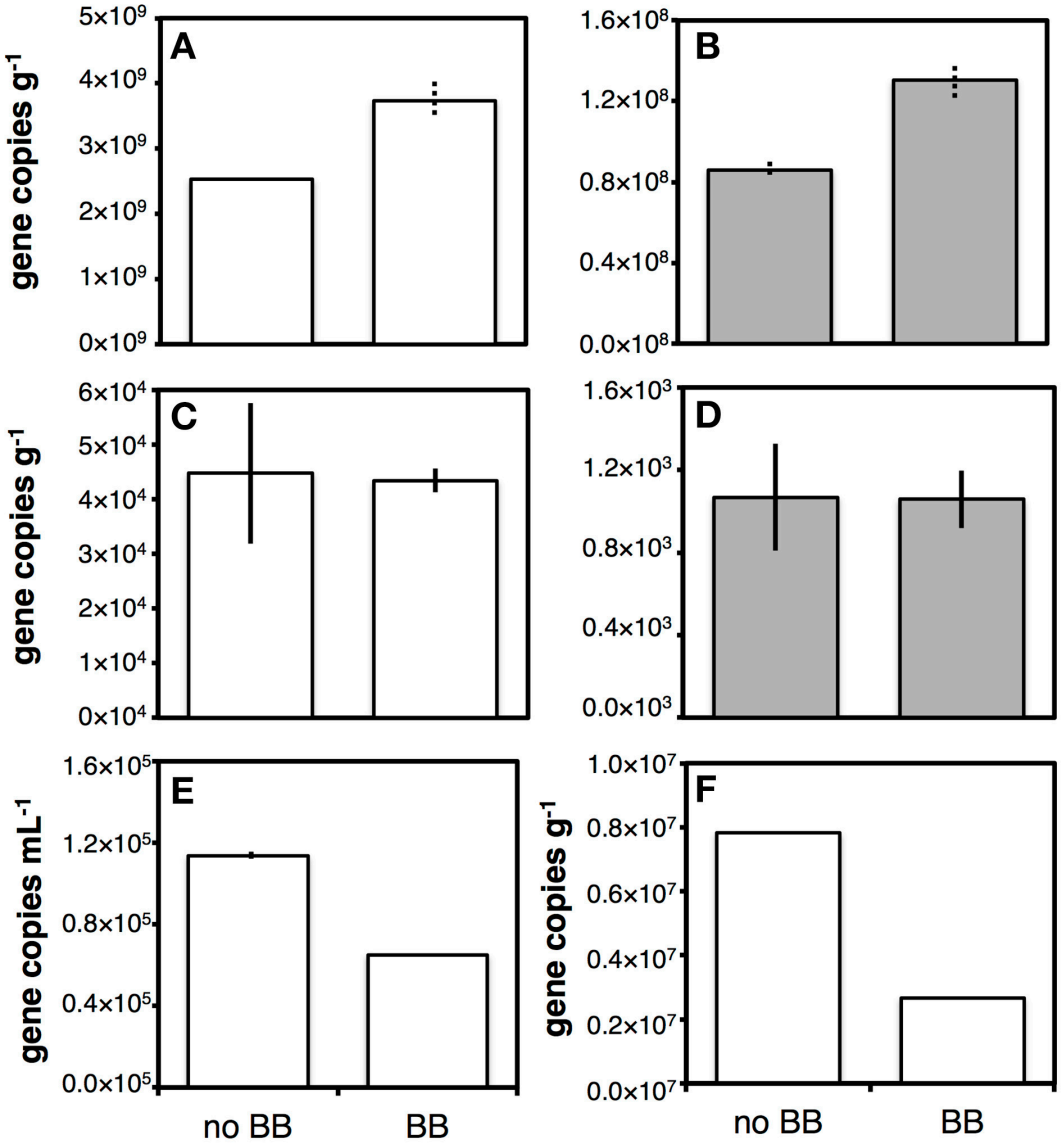
**Bar chart illustrating the effect of bead-beating on DNA yield determined by qPCR of 16S rRNA genes**. BB indicates sample was bead-beaten prior to freeze-thawing and chemical lysis, no BB indicates no bead-beating prior to freeze-thawing and chemical lysis. Chemical lysis consisted of incubating samples in a lysis buffer with guanidium hydrochloride and pH 10 for 1 h at 50°C (see Section on Chemical/Enzymatic Lysis). White bars indicate bacterial copy numbers, gray bars archael copy numbers. Tests were performed on Aarhus Bay station M1 sediment **(A,B)**, Subglacial Lake Whillans sediment **(C,D)**, drilling mud **(E)**, and subseafloor sediment cuttings **(F)**. Only bacterial qPCR checks were performed on drilling mud and cuttings. Solid error bars indicate ranges of two replicate extractions, dashed error bars indicate ranges of PCR replicates on the same extract.

We compared effects of bead-beating on gene copy numbers across organic-rich coastal sediment, oligotrophic subglacial lake sediment, drilling mud, and subseafloor sediment cuttings (Figure 1). Effects varied, with bead-beating increasing bacterial and archaeal 16S rRNA gene copy numbers by ~50% in coastal sediment (Figures 1A,B). No effect was observed by contrast in subglacial lake sediment (Figures 1C,D). In drilling mud and sediment cuttings bead-beating lowered bacterial gene copy numbers by ~40 and ~65%, respectively (Figures 1E,F).

#### Homogenizer

Spillage and overheating of sediment extracts occurred during the use of homogenization probes. Overheating also occurred when these probes were directly applied to basalt rock. We conclude that this physical disruption method is not compatible with our extraction method.

#### Chemical/enzymatic lysis

Chemical and enzymatic lysis treatments consisted of incubating samples in lysis solutions for 1-h cycles at 50°C and gentle shaking. Typically these cycles were preceded by freeze-thawing. The combination of freeze-thawing and 1-h incubations at 50°C is termed a freeze-thaw+heat cycle.

#### Effects of enzymes and SDS, test I

**Figure 2:**
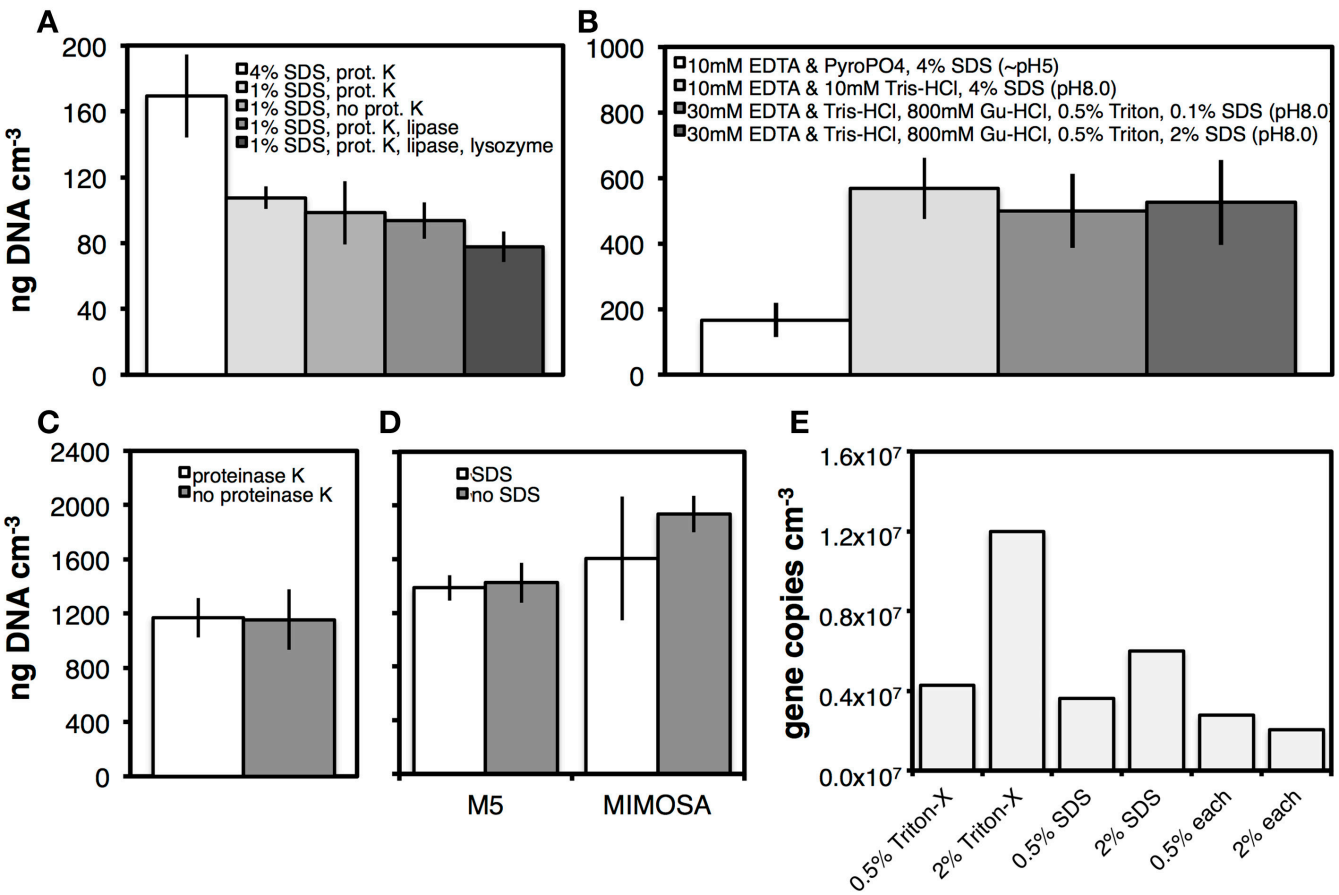
**Effects of lysis buffer composition on DNA yields. Sediment from Aarhus Bay Station M5 was used in (A–D)**. In addition, sediment from Aarhus Bay Station MIMOSA was used in **(D)**. Drilling mud was used for the tests shown in **(E)**. **(A)** Effect of two SDS concentrations (4%, 1%) and presence of three different enzymes on DNA yields in a pyrophosphate (pyroPO_4_)-based DNA extraction buffer. **(B)** Effects of DNA extraction buffer composition on DNA yields (Gu-HCl, guanidium hydrochloride). **(C)** Effect of proteinase K on DNA yields using a Tris-EDTA-based extraction buffer containing guanidium hydrochloride and Triton X-100. **(D)** Effect of SDS (0.1%) on DNA yields wity a Tris-HCl–EDTA (TE)-based extraction buffer containing guanidium hydrochloride and Triton X-100. **(E)** Comparison of DNA yields at two concentrations of Triton X-100 and SDS. The two detergents were added alone or in combination to the TE-based extraction buffer containing guanidium hydrochloride. Error bars in **(A–D)** indicate the standard deviations of three replicate DNA extractions. Enzyme concentrations were 1 μg mL^−1^ of extract.

DNA yields with SDS and lysis-enhancing enzymes were compared. Using an extraction solution of 3% NaCl, 10 mM EDTA, 10 mM sodium pyrophosphate, and 0.1% Tween 80, SDS concentration had a significant effect on DNA yields (*p* < 0.05). In lysis incubations that included proteinase K, yields obtained with 4% SDS were 60% higher than yields with 1% SDS (Figure 2A). Omission of proteinase K had no significant effect on DNA yields at 1% SDS. Similarly, neither the combination of proteinase K and lipase nor the combination of proteinase K, lipase, and lysozyme increased DNA yields relative to treatments where enzymes were omitted.

#### Chemical composition of extraction buffer

We kept the proteinase K treatment and examined how changes in extraction buffer composition affected DNA yield. The variables tested were pH (5.0 vs. 8.0), SDS concentration (0.1–4%), addition of the chaotropic compound guanidium hydrochloride (800 mM), and addition of the nonionic membrane-disrupting detergent Triton X-100 (0.5%) (Figure 2B). Parallel to increasing the pH from 5.0 to 8.0, we changed from a phosphate buffer to a Tris-HCl buffer. This change in buffer composition and pH significantly (*p* < 0.01) increased the DNA yield. Adding guanidium hydrochloride and Triton X-100 had no significant effect, and neither did reducing the SDS concentration from 4 to 0.1% in buffer containing guanidium hydrochloride and Triton X-100.

#### Effects of enzymes and SDS, test II

The results in Figure 2A suggested that proteinase K might not increase DNA yields in the presence of SDS, while the results in Figure 2B indicated that DNA yields did not depend on SDS concentration in the presence of guanidium hydrochloride and Triton X-100. To investigate this further, we continued our tests using the same guanidium hydrochloride and Triton X-100 containing buffer (30 mM EDTA, 30 mM Tris-HCl, 800 mM guanidium hydrochloride, 0.5% Triton X-100, pH 8.0) and examined possible benefits of proteinase K and SDS addition. As before, proteinase K did not increase DNA yields from M5 sediment (Figure 2C). Moreover, in treatments without proteinase K, addition of SDS to lysis solution containing guanidium hydrochloride and Triton X-100 did not increase DNA yields from the M5 or MIMOSA sites (Figure 2D), contrary to its DNA yield enhancing effect in the absence of guanidium hydrochloride and Triton X-100 (Figure 2A). We additionally compared treatments with and without SDS by bacterial and archaeal qPCR assays. These indicated negative effects of SDS, with significantly higher (*p* < 0.05) copy numbers of Bacteria in extractions from M5 and MIMOSA and Archaea in extractions from M5 where SDS had been omitted (Figures S1A,B). As a final trial, we used samples of drilling mud as test material and examined DNA yields using extraction buffer amended with (a) Triton X-100 but no SDS (b) SDS but no Triton X-100, or (c) both Triton X-100 and SDS, using bacterial 16S rRNA gene copy numbers as an indicator of yield (Figure 2E). For both detergents, we observed positive concentration-dependent effects when they were added alone. Yet, Triton X-100 produced higher bacterial copy numbers when the same volumes of detergent were added (0.5%, 2%). By contrast, adding both detergents in combination negatively affected DNA yields, with the lowest bacterial copy numbers in treatments with 2% of each Triton X-100 and SDS. Interestingly, increasing the amount of Triton X-100 from 0.5 to 2% lowered the DNA yield from sediment samples (data not shown). Thus, we—contrary to the results based on drilling mud—opted for a final lysis solution consisting of 30 mM Tris-HCl, 30 mM EDTA, 800 mM guanidium hydrochloride, and—unless specified otherwise—0.5% Triton X-100. We termed this solution lysis solution I.

#### CTAB/PVPP

We investigated possible benefits of adding a second aqueous lysis solution, consisting of 2.5 M NaCl, CTAB, and/or PVPP. This second lysis solution was added after samples had undergone two freeze-thaw+heat cycles with lysis solution I. After adding the second lysis solution, samples underwent two more freeze-thaw+heat cycles, however, the temperature during the final heat incubation was raised to 65°C. The purpose of the 65°C cycle was to induce binding of CTAB to polysaccharides and subsequent removal of polysaccharides during centrifugation. The high NaCl concentration was chosen so that CTAB—which binds to DNA at concentrations below 0.7 M (Murray and Thompson, 1980)—did not remove DNA. PVPP was added because it binds polyphenolic substances. Addition of this solution reduced the color of the supernatant, and slightly, but not significantly increased DNA yields from organic-rich sediment from Aarhus Bay, when 2% CTAB and 0.1% PVPP were applied (Figure S1C). We termed this solution lysis solution II.

#### Reductants

To prevent DNA oxidation reactions during extractions and increase DNA recovery by breakage of disulfide bonds, we tested adding the reductants β-mercaptoethanol and TCEP to lysis solution I. However, neither chemical significantly affected DNA yields (data not shown).

#### Comparison of lysis protocols (LPs)

Over the course of our tests, three different chemical lysis protocols were established, each suited for a different sample type or extraction criterion. Lysis Protocol I (LP I) had the shortest lysis protocol, which consisted of bead-beating in the presence of lysis buffer I, phosphate solution, and PCI. Inclusion of PCI had been shown to greatly enhance DNA yields during rapid lysis (Figure S2A). Lysis Protocol II (LP II) consisted of typically one, but in some cases up to three, freeze-thaw+heat cycles in the presence of lysis buffer I and phosphate solution (but not PCI), which were in some cases preceded by bead-beading. Lysis Protocol III (LP III) was the most extensive protocol, consisting of two freeze-thaw+heat cycles in lysis buffer I and phosphate solution, followed by two more freeze-thaw+heat cycles after lysis buffer II addition, with the final 1-h incubation at 65°C. Samples processed by LP III were typically not bead-beaten.

**Figure 3:**
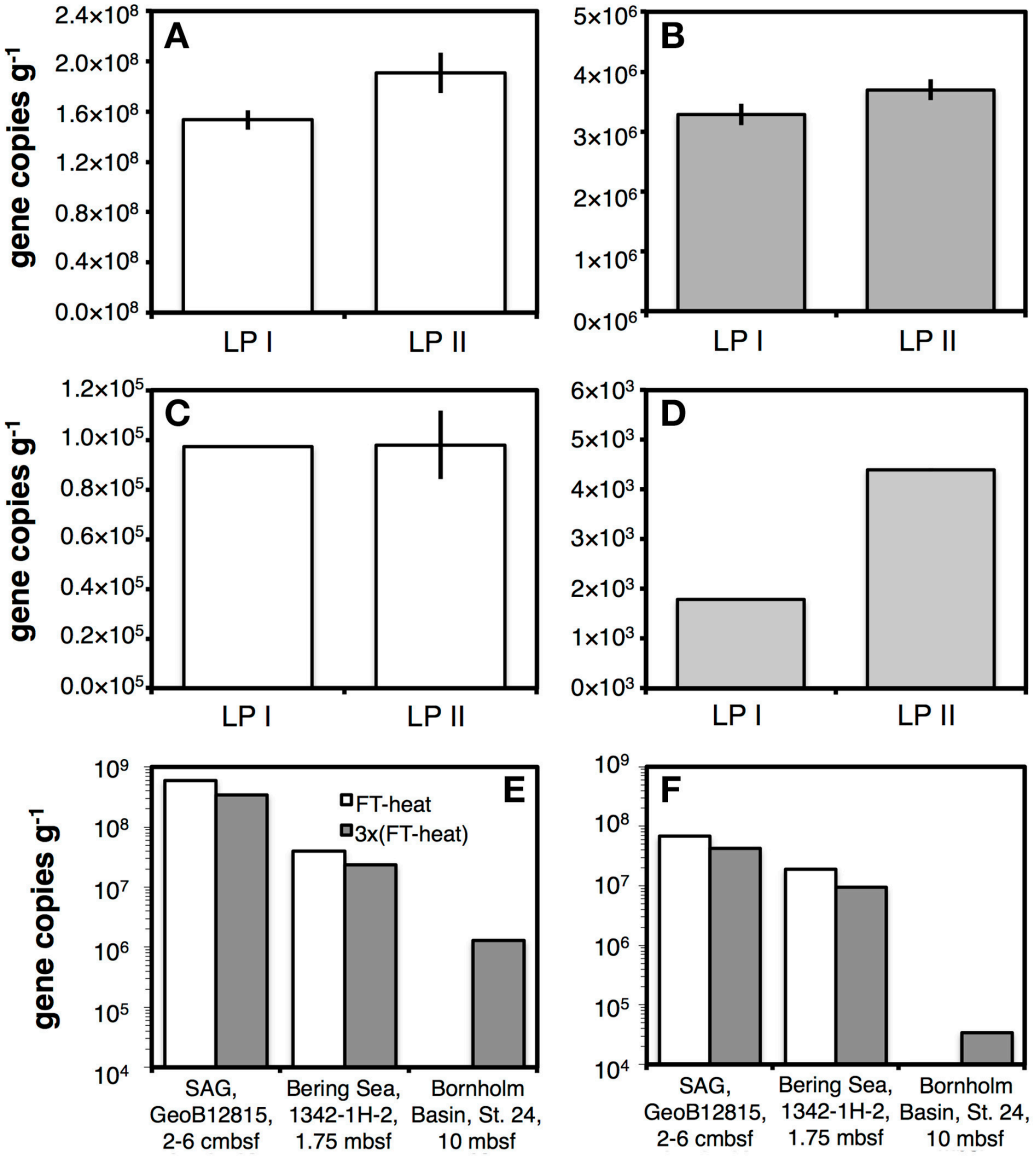
**Results of qPCR assays on bacterial 16S rRNA gene copy numbers are shown in the left column, results for Archaea in the right column. (A,B)** Gene copy numbers in sediment from an oligotrophic glacial lake in Greenland obtained using a rapid lysis method involving PCI addition during bead-beating and no freeze-thaw+heat cycles (LP I) compared to a slower lysis method involving bead-beating without PCI followed by one freeze-thaw+heat cycle (LP II). **(C,D)** Same treatments as in **(A,B)**, tested on sediment from highly oligotrophic Subglacial Lake Whillans. **(E,F)** Effects of increasing the number of freeze-thaw+heat (FT-Heat) cycles from one to three on oligotrophic sediments from three locations. Error bars indicate data ranges for samples where extractions were duplicated **(A–D)**.

When comparing DNA yields obtained with LPs I and II, marginal increases in bacterial and archaeal 16S rRNA gene copy numbers occurred in glacial lake sediment treated with LP II compared to LP I (Figures 3A,B). For sediment from Subglacial Lake Whillans, archaeal copy numbers increased more than twofold compared to LP I (Figure 3D), but there was no difference in bacterial copy numbers (Figure 3C). In certain highly oxidized samples, e.g., pelagic red clay from the South Pacific Gyre, the use of phenol during LP I was not suitable, as revealed by a color change from clear to red after bead-beating, which indicated pronounced phenol oxidation, possibly by iron oxides.

When comparing DNA yields obtained with LP II and LP III across different sample types, we found that LP III was typically not suitable for applications with organic-poor, low-biomass samples, as revealed by consistently lower DNA yields (Figure S2B,C). We thus tested effects of additional freeze-thaw+heat cycles as part of LP II—rather than including lysis solution II as in LP III—on oligotrophic sediments from three locations. In surface sediment from the South Atlantic Gyre and shallow subsurface sediment of the Bering Sea (IODP Site 1342), bacterial and archaeal gene copy numbers were decreased twofold by additional freeze-thaw+heat cycles. However, bacterial and archaeal gene copy numbers from buried ice lake clay of the Bornholm Basin were increased above detection by these additional freeze-thaw+heat cycles (Figures 3E,F).

#### Adsorption prevention

##### pH

**Figure 4:**
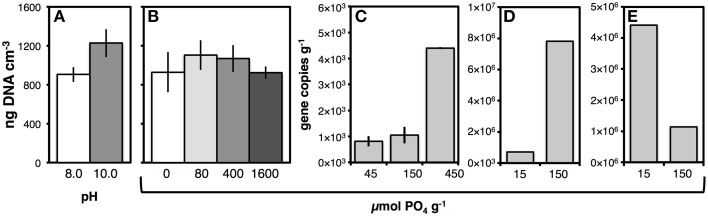
**(A)** Effect of pH of lysis buffer I on DNA yields from sediment of Aarhus Bay Station M5. **(B)** Effect of adding different amounts of pyrophosphate on DNA yields from Aarhus Bay Station M5. The PO_4_ amounts shown correspond to 0, 40, 200, and 800 μmol pyrophosphate g^−1^ sediment. **(C–E)** Effect of adding different amounts of dNTPs—expressed in PO_4_ monomer units—on extracted archaeal gene copies g^−1^ from sediment of Subglacial Lake Whillans **(C)**, and bacterial gene copies g^−1^ from sediment cuttings **(D)** and drilling mud **(E)**. Error bars in **(A,B)** indicate standard deviations of triplicate DNA extractions. Error bars in C indicate data ranges of duplicate DNA extractions.

We tested whether DNA yields from station M5 would increase by raising the pH of lysis solution I from 8.0 to 10.0, and measured a ~30% increase in DNA yields (Figure 4A; *p* < 0.05). From then on, a pH of 10.0 was used in lysis solution I.

##### Phosphate addition

We tested how adding different phosphate species prior to cell lysis affected nucleic acid recovery. Phosphate species tested included pyrophosphate, hexametaphosphate, dNTPs, a 30-bp PCR product consisting of double-stranded DNA, and salmon sperm double-stranded DNA. In organic-rich sediment from Aarhus Bay Station M5, addition of pyrophosphate prior to cell lysis caused an average increase in DNA yield at intermediate phosphate additions, but this effect was not statistically significant (Figure 4B). There was no difference in the effect of pyrophosphate and hexametaphosphate on DNA yields from M5 or Aarhus Bay Station M1 (Figure S3A). Similarly, adding two different concentrations of dNTPs to Greenland glacial lake sediment had no effect on bacterial or archaeal gene copies (Figure S3B). This changed when dNTPs were added as a PO_4_ source to oligotrophic sediment from Subglacial Lake Whillans. While increasing the PO_4_ treatment from 45 to 150 μmol g^−1^ sediment only resulted in a slight increase in archaeal gene copies, further increasing the PO_4_ dose to 450 μmol g^−1^ increased archaeal copy numbers by a factor of five, from 8.2 × 10^2^ copies g^−1^ sediment at 45 μmol PO_4_ g^−1^ sediment to 4.4 × 10^3^ copies g^−1^ sediment at 450 μmol PO_4_ g^−1^ sediment (Figure 4C). These elevated archaeal gene copy numbers at the high PO_4_ treatment were reproduced in a second extraction test (5.3 × 10^3^ copies g^−1^ sediment; Figure S3C), and the same PO_4_-concentration-dependent increase in copy numbers was seen when the rapid lysis method with PCI was employed, albeit at overall lower copy numbers (data not shown). Similarly, increasing the amount of PO_4_ dose from 15 to 150 μmol g^−1^ sediment cuttings resulted in an 8-fold increase in DNA yields (Figure 4D). Yet, the opposite trend, i.e., lower DNA yield at high PO_4_ addition was also observed. Drilling mud had ~75% lower DNA yields, when amended with 150 μmol g^−1^ PO_4_ instead of only 15 μmol g^−1^. DNA yields in several other samples, e.g., sediment from Guaymas Basin and off Greenland, were also markedly higher at low PO_4_ additions (data not shown). By comparison, addition of small amounts of dsDNA (0.001 μmol PO_4_ g^−1^ sediment) did not increase archaeal or bacterial gene copy numbers (Figure S3C). We also tested addition of salmon sperm DNA. The results were inconclusive, however, as viewing qPCR products on agarose gels revealed unspecific amplifications of both bacterial and archaeal 16S rRNA genes. This suggested that—without further treatment of salmon sperm DNA to eliminate this unspecific amplification—reliable quantifications of bacterial and archaeal gene copy numbers were not possible.

#### Purification

After the various lysis and adsorption prevention treatments, we washed nucleic acid extracts with CI or PCI. Initially, we had also tested washes with phenol and chloroform; however, DNA yields were lower with pure phenol, and chloroform alone failed to produce a sharp interface between the aqueous and organic phase. In the absence of a sharp interface, clean transfers of aqueous supernatants containing nucleic acids were more difficult. Due to the stabilizing effect of isoamylalcohol on aqueous-organic interfaces, we only used CI or PCI from then on.

**Figure 5:**
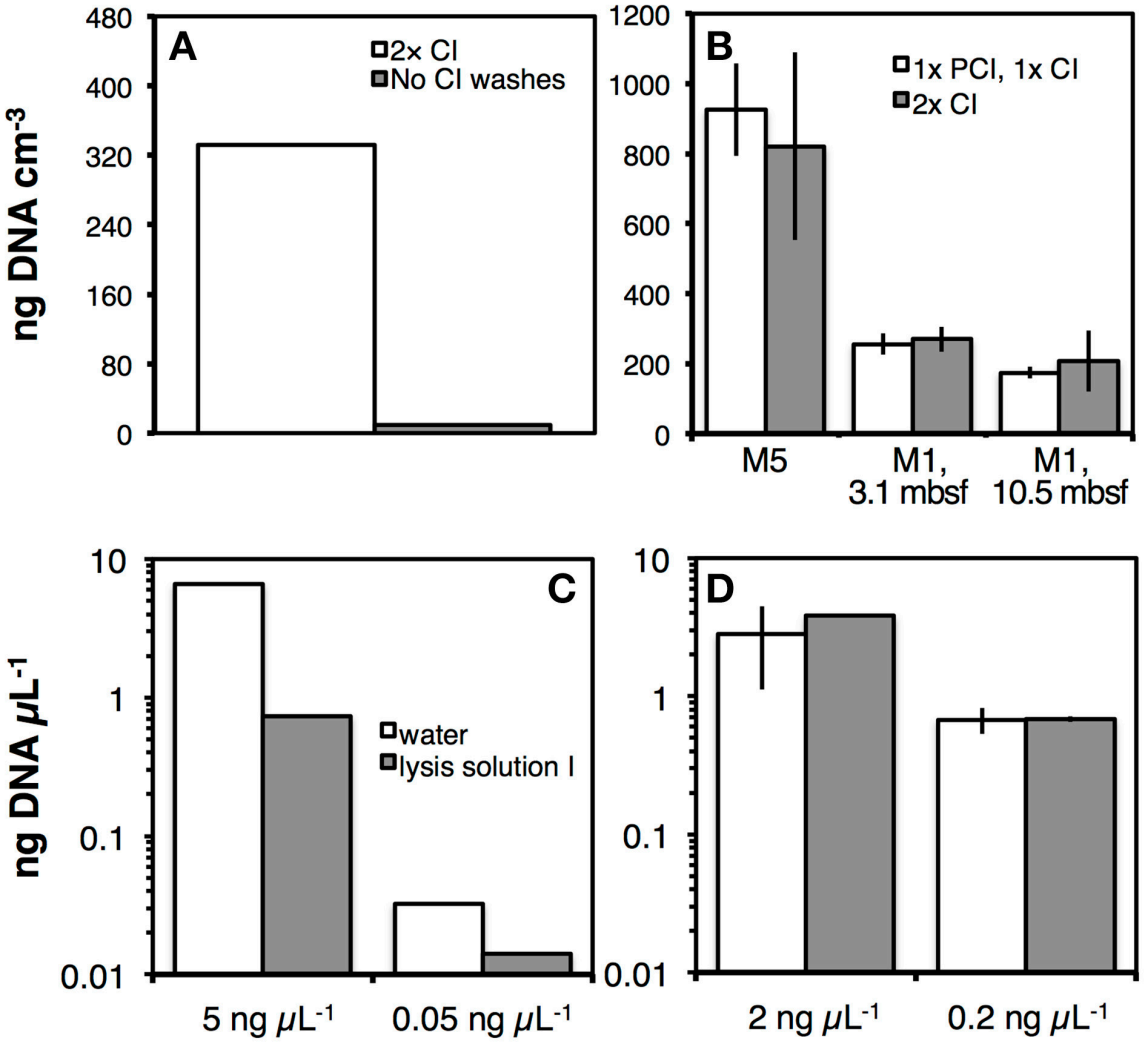
**(A)** Effect of two CI washes on DNA recovery from Aarhus Bay station M5 sediment after extraction using LP III. **(B)** Comparison of DNA recovery from samples that were purified by one PCI wash, followed by one CI wash to samples that were purified by two CI washes. Samples were from marine sediments of Aarhus Bay Stations M5 and M1 (3.1 mbsf), and from a deeply buried terrestrial soil layer at Station M1 (10.5 mbsf). **(C,D)** Effect of chloroform washes on DNA ladder recovery. In two separate experiments, two different concentrations of 100-bp DNA ladder were prepared by dilutions with water or lysis solution I. These ladder solutions were directly precipitated in PEG 8000-NaCl solution **(C)** or washed twice with CI and then precipitated in PEG 8000-NaCl **(D)**. The resulting DNA pellets were dissolved in TE buffer and quantified spectrofluorometrically. Error bars indicate standard deviations of tests that were run in triplicate.

The importance of washing with CI or PCI after the previously described lysis treatments is evident from Figure 5. In DNA extractions following a precursor of LP III, that still involved pH 5 extraction buffers with SDS and proteinase K treatment (Figure 2A), omission of CI washes resulted in dramatic downstream losses of DNA (Figure 5A). DNA yields after extraction following the final LP III did not differ significantly if DNA extracts were washed two times with CI or once with PCI followed by once with CI (Figure 5B). Several observations were made nonetheless: on one hand, washes with PCI more efficiently removed color, detergent and precipitates than the initial CI wash. Thus, in some cases, three CI washes were necessary to remove precipitates and obtain the same visual purity as after one PCI and one CI wash. On the other hand, phenol oxidation, indicated by pinkish to bright red color, often occurred after vortexing PCI with DNA extracts. This discoloration was—as mentioned earlier—most prominent in oxidized sediments, such as red clays. However, even in organic-rich, anoxic sediment from Aarhus Bay Station M1 there was an increase in phenol oxidation, from being virtually absent in surface sediments to being pronounced in deeper layers, especially with extracts from the terrestrial soil layer. To reduce phenol oxidation, we tested adding the reductants 2-hydroxyquinoline, β-mercaptoethanol, or TCEP to PCI, or to lysis solution 1, however, none of these reductants visibly reduced phenol oxidation.

In further tests, we examined effects of lysis solution I carryover on DNA recovery. We used DNA ladder instead of DNA extracts as a template, and mixed this ladder with water or lysis solution I prior to precipitation with polyethylene glycol 8000 solution. The results show that in the absence of CI washes chemicals present in lysis solution I dramatically lower DNA recovery (Figure 5C). This problem is eliminated by two CI washes prior to DNA precipitation (Figure 5D).

#### Precipitation

**Figure 6:**
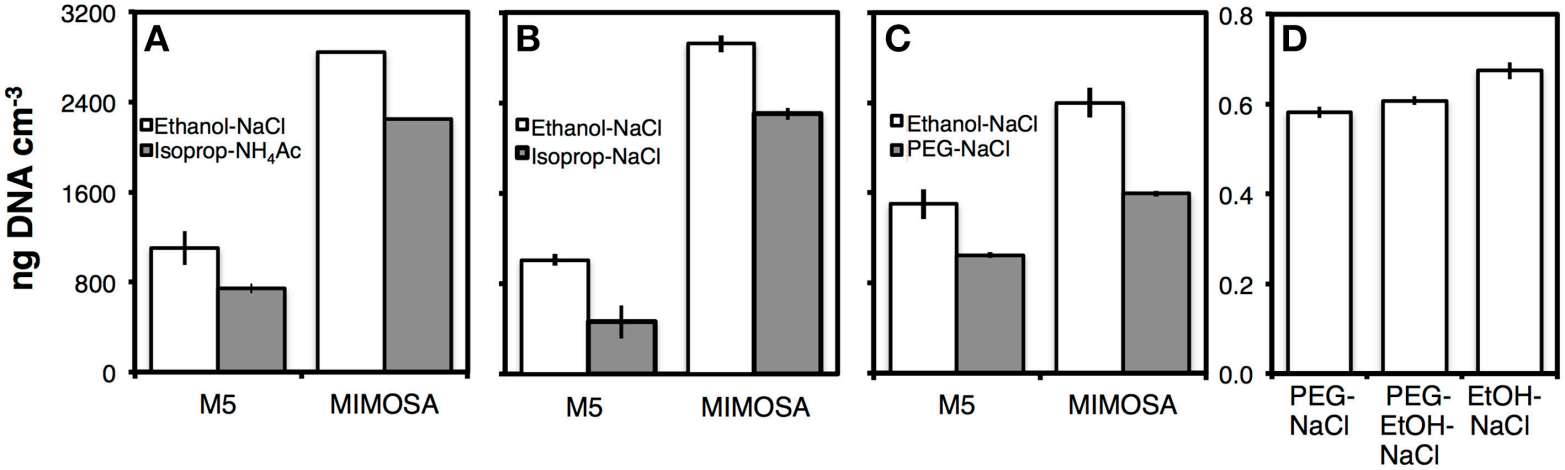
**Comparisons of DNA yields by different precipitation methods in relation to Ethanol-NaCl precipitation. (A)** Isopropanol-ammonium acetate precipitation; **(B)** isopropanol-NaCl precipitation; **(C)** PEG 6000-NaCl precipitation; **(D)** PEG 8000-NaCl and PEG-EtOH-NaCl precipitation. As a co-precipitant, we added LPA to a concentration of 20 μg mL^−1^ of extract to all samples shown in **(A–D)**. LPA and salt solutions were homogenized with extracts prior to adding alcohol or PEG solution. This ensured that nucleic acids in solution were exposed to LPA and added salt. Without this prior homogenization step, LPA was immediately precipitated without going into solution. Due to light sensitivity of LPA, all precipitations were for 2 h in the dark. **(A,B)** were performed at −20°C **(C,D)** at room temperature. Error bars in **(A-C)** indicate standard deviations of tests that were run in triplicate, error bars in **(D)** indicate data ranges of tests that were run in duplicate.

We compared DNA yields after precipitation with Ethanol-NaCl to precipitations with Isopropanol-Ammonium Acetate (Figure 6A), Isopropanol-NaCl (Figure 6B), PEG 6000-NaCl (Figure 6C), PEG 8000-NaCl, or PEG 8000-Ethanol-NaCl (both Figure 6D). In all comparisons, DNA yields were significantly higher after Ethanol-NaCl precipitation (*p* < 0.05). In comparison to Ethanol-NaCl precipitation, Isopropanol-Ammonium Acetate precipitation resulted in ~20–30%, Isopropanol-NaCl in ~20–50%, and PEG 6000-NaCl in 30–40% lower DNA yields. PEG 8000-NaCl and PEG 8000-Ethanol-NaCl performed the next best with only ~10% lower DNA yields.

We also checked the influence of precipitation temperature—i.e., room temperature vs. −20°C—and possible benefits of higher NaCl concentration—i.e., 1.2 M vs. 1.8 M prior to ethanol addition—on DNA yields. Neither temperature nor salt concentration improved DNA yields (Figures S4A,B). DNA pellets were smallest when precipitation was conducted at room temperature, possibly due to less co-precipitation of residual detergent, and with the lower NaCl concentration, due to less co-precipitation of salt. These smaller pellets more readily dissolved after drying. Consequently, we opted for precipitations at room temperature and 1.2 M NaCl concentrations.

The size of DNA pellets differed markedly between different precipitation methods. Ethanol-NaCl produced the largest and PEG-NaCl produced the smallest pellets. In fact, after PEG precipitation, DNA pellets were frequently invisible and often did not stick to centrifuge tube walls. In ~10–30% of the cases, significant fractions or most of the DNA pellet were lost during the removal of PEG-NaCl solution or subsequent wash steps with 80% ethanol. The solution was to avoid decanting and to remove supernatants as follows: tubes were held vertically, supernatants were slowly pipetted off from the surface downward, with the pipet tip closely following the liquid-air interface, and pipetting was stopped when ~20 μL of liquid were left in the tube. By pipetting in this fashion, no DNA pellets were lost any longer after precipitations with PEG 8000-NaCl or PEG 6000-NaCl and final DNA yields were only ~10% lower than after ethanol-NaCl precipitation.

To further improve our precipitation methods, we examined whether interactions between the amount of PO_4_ added and the precipitation method affected the DNA yield and purity after precipitation (Figure S4C). Based on 1:10 dilutions of extracts, there was no difference in bacterial 16S rRNA gene copy numbers at different amounts of PO_4_ added (15 vs. 150 μmol PO_4_ g^−1^ sediment) or using different precipitation methods (PEG-NaCl, PEG-EtOH-NaCl, EtOH-NaCl). However, based on undiluted extracts, PCR inhibition was more pronounced in high PO_4_ treatments, and highest in high PO_4_ treatments that had been precipitated with ethanol. We also tested PEG solutions with different NaCl concentrations (2 M, 1.2 M, 0.8 M, 0.4 M) but detected no improvement in DNA recovery compared to 1.6 M NaCl (data not shown). In further tests on the PEG-NaCl precipitation method, this time using dilutions of DNA ladder as test material, we observed a clear detrimental effect of autoclaving PEG 8000-NaCl (Figure S4D). Furthermore, effects of centrifugal force (14,000 vs. 20,000×*g*), adding MgCl_2_ or acetate, or lowering the pH of PEG solution were examined (Figure S4E). With overall DNA recovery being very high and virtually no DNA loss compared to non-precipitated original solutions, none of these treatments performed significantly better than the others.

#### Final purification

**Figure 7:**
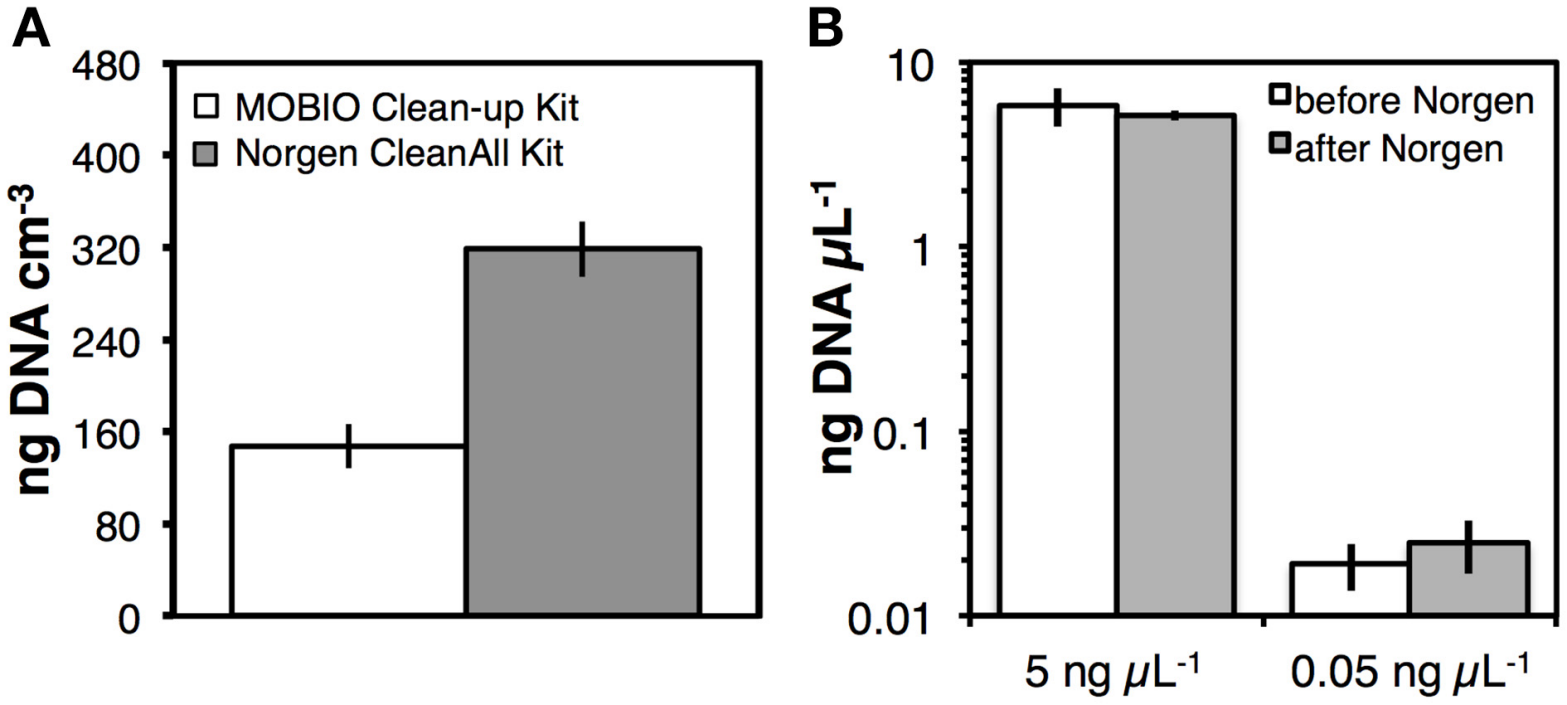
**(A)** Comparison of DNA recovery using two commercial kits, the PowerClean DNA Clean-Up Kit (MO BIO Laboratories), and the Clean All RNA/DNA Clean-up and Concentration Kit (Norgen Biotek). These kits were used to further purify DNA after Ethanol-NaCl precipitation. Triplicate DNA extracts from Station M5 were divided into equal parts for these tests, with each half of the extract purified by a different kit. **(B)** Comparison of DNA ladder concentrations before and after cleanup with the kit by Norgen Biotek. Error bars indicate standard deviations of tests that were run in triplicate.

Though PCR amplification without further purification was in some cases possible after nucleic acids had been precipitated, washed twice with 80% ethanol, air-dried, and subsequently dissolved in water or TE buffer (Figure S4C), PCR inhibition of undiluted DNA extracts was common. Since extract dilution reduces the detection sensitivity of nucleic acids by spectrofluorometry or qPCR. we tested commercial DNA purification kits, i.e., the PowerClean DNA Clean-Up Kit (MO BIO Laboratories), and the CleanAll RNA/DNA RNA/DNA Clean-up and Concentration Kit (Norgen Biotek). Both kits resulted in clean DNA extracts, where PCR inhibition was absent when undiluted DNA extracts were used as PCR templates. Yet, we observed a ~50% lower DNA yield after purification with the PowerClean compared to the CleanAll RNA/DNA kit (Figure 7A; *p* < 0.05). Further tests to examine DNA recovery using two concentrations of DNA ladder indicated no significant DNA loss using the CleanAll RNA/DNA kit at both ladder concentrations (Figure 7B).

### Separation of DNA pools

We developed a protocol to separately extract sDNA and nsDNA. sDNA is initially separated from the nsDNA using a wash step. Subsequently, the same general protocol that was the outcome of the tests outlined in the previous section was used.

#### sDNA extraction protocol

The sDNA fraction is extracted by a 1-h incubation of 0.2 g sediment with carbonate dissolution mix (CDM; 0.43 M acetic acid, 0.43 M sodium acetate, pH 4.7—composition based on Kallmeyer et al. (2008)—and sufficient PO_4_ to minimize DNA sorption) followed by a second 1-h incubation after addition of 10× TE buffer (300 mM Tris-HCl, 10 mM EDTA, pH 10.0). Throughout the incubations, samples are kept at room temperature and gently mixed, e.g., using a shaker incubator or rotator mixer (600 rpm). The NaCl concentration in the CDM and TE buffers is adjusted to reflect the salinity of samples, e.g., 3% NaCl for typical seawater samples. After the second hour of incubation, samples are centrifuged for 20 min at 10,000×g. The supernatant containing sDNA and the sediment pellet containing nsDNA are then separated and subsequently undergo separate treatments, which closely follow those of the “General Protocol.”

#### Desorption of sDNA

**Figure 8:**
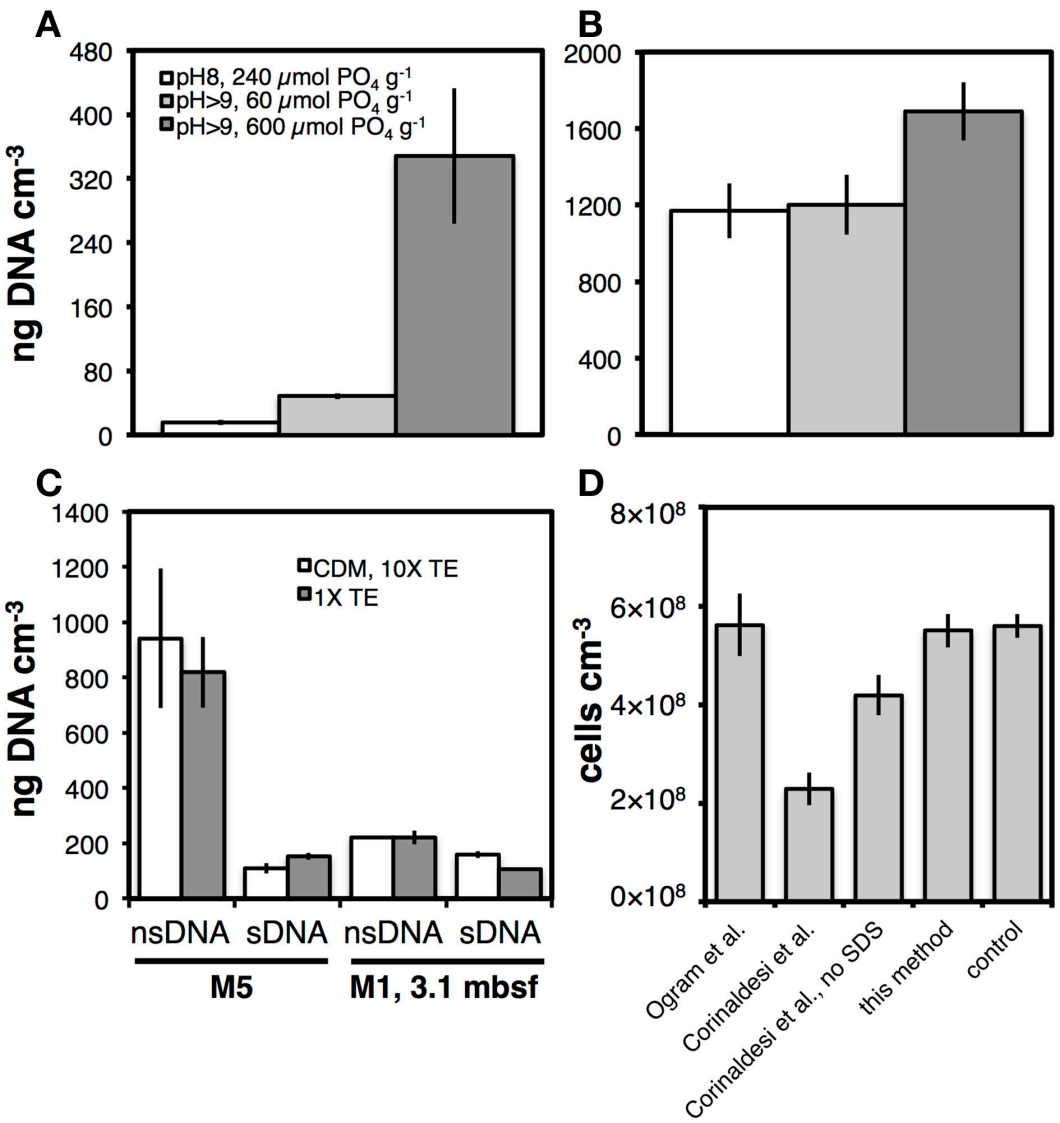
**Effect of pH and amount of PO_4_, added as hexametaphosphate, g^−1^ sediment on the yield of the **(A)** sDNA fraction, and (B) nsDNA fraction from the same samples from Aarhus Bay Station M5 (legend in A also applies to B)**. The pH was controlled by the ratios of CDM and 10× TE added: a pH of 8 was produced by adding 800 μL CDM and 800 μL of 10× TE, a pH > 9 by adding 200 μL CDM and 1600 μL 10× TE. Hexametaphosphate was added with the CDM, and concentrations in the CDM were 10 mM in the 240 and 60 μmol PO_4_ treatments and 100 mM in the 600 μmol PO_4_ treatment. **(C)** Effect of two different sDNA extraction protocols on sDNA and nsDNA yields from Aarhus Bay Stations M5 and M1. The first protocol (CDM, 10× TE) is equivalent to the third protocol (pH > 9, 600 μmol PO_4_ g^−1^) in **(A,B)**. The second protocol (1× TE) consisted of incubating 0.2 g of sediment with 1800 μL of 1× TE buffer at room temperature and gentle shaking at 600 rpm for 2 h. The 1× TE buffer had been corrected for salinity and amended with the same amount of hexametaphosphate as introduced by the CDM solution (30 mM Tris-HCl, 1 mM EDTA, 3% NaCl, 11 mM metaphosphate, pH 10.0). **(D)** Comparison of cell counts on sediments that had undergone different methods of sDNA extraction. These methods were by Ogram et al. (1987), Corinaldesi et al. (2005), without SDS, and *this method*. Controls consisted of sediment that had not undergone sDNA extraction. Note: Results shown for M5 in **(A,B)** are from a different extraction trial than those shown for M5 treated with CDM, 10× TE in **(C)**. Error bars indicate standard deviations of tests that were run in triplicate.

We examined the effects of pH, amount of PO_4_ added g^−1^ sediment, and sDNA extraction solution composition on the yields of sDNA and nsDNA (Figures 8A,B). For sediment from Aarhus Bay Station M5, the highest yields of sDNA and nsDNA were obtained when a high amount of PO_4_ was added along with the CDM, and the pH during the final hour of sDNA extraction exceeded 9 (Figure 8A). At the pH and PO_4_ range tested, pH was the most important variable, with a threefold increase in sDNA recovery resulting from raising the pH from pH 8 to pH > 9 in spite of a fourfold lower amount of PO_4_ added (*p* < 0.01). Yet, increasing the PO_4_ dose from 60 to 600 μmol g^−1^ at pH>9 contributed an additional 5-fold increase in sDNA yield (*p* < 0.05), and demonstrated that both pH and PO_4_ dose are critical variables in maximizing sDNA yields. By comparison, the effects of pH increase and PO_4_ reduction either canceled each other out or had no effect on the nsDNA yield (Figure 8B). Only the combination of pH > 9 and high PO_4_ resulted in a significant (~40%; *p* < 0.05) increase in nsDNA yield.

#### sDNA extraction buffer composition

We investigated effects on sDNA and nsDNA yields of changing from the 1-h treatment with CDM followed by a 1-h treatment with 10× TE to a 2-h treatment with only 1× TE (30 mM Tris-HCl, 1 mM EDTA, PO_4_ dose as previously in CDM, pH 10.0; Figure 8C). No effect was seen. We also checked for cell lysis as a result of the CDM-10× TE treatment, by performing cell counts on sediment pellets after sDNA extraction (Figure 8D). There was no difference in cell numbers after our sDNA extraction protocol compared to controls without sDNA extraction. To assess how other sDNA extraction methods compared to ours, we also performed cell counts on sediments after sDNA extraction following the protocols by Ogram et al. (1987) and Corinaldesi et al. (2005). We observed no cell loss after treatment by the Ogram et al. method, but the cell recovery after treatment as in Corinaldesi et al., was only ~40%. Further tests, in which we omitted SDS, which had been included at low concentration in the original sDNA extraction method by Corinaldesi et al., revealed that ≥50% of the cell lysis in this protocol was due to SDS.

We then examined possible sources and particle size-associations of sDNA. The first test was to check whether sDNA consisted of cells that had remained in solution after 20 min of centrifugation at 10,000×g. Our results indicate that nearly all sDNA passes through a 0.2 μm-pore size filter (Figure S5A), and that in most samples the bulk of sDNA was free or attached to particles smaller than 0.02 μm (Figure S5B). Ratios of the amount of DNA passing through 0.02 μm relative to the amount of DNA in the 0.02–0.2 μm size fraction varied from 2:1 to 10:1 in marine sediment layers (0.05–3.1 mbsf). In a buried terrestrial soil layer (10.5 mbsf) this ratio was, however, inverted, with 3 times more DNA in the 0.02–0.2 μm size fraction than in the <0.02 μm size fraction. The same quantitative trends in DNA size fractions were confirmed by qPCR assays on 16S rRNA genes (Figures S5C,D).

We then examined if repeated freezing of sediment samples affected the extracted nsDNA pool size (Figure S6). One-time freezing had virtually no effect on the size of the nsDNA pool. Even two freeze-thaw cycles yielded no statistically significant change in extracted nsDNA.

We conclude these tests by examining size distributions of sDNA and nsDNA pools by gel electrophoresis, using two different precipitation and cleanup methods (Figure S7). Our results show no difference in DNA size distributions for nsDNA. However, sDNA precipitation with ethanol followed by purification via the Norgen CleanAll RNA/DNA kit resulted in a strong bias against short DNA fragments compared to PEG precipitation without subsequent cleanup.

### Simultaneous extraction of DNA and RNA

**Figure 9:**
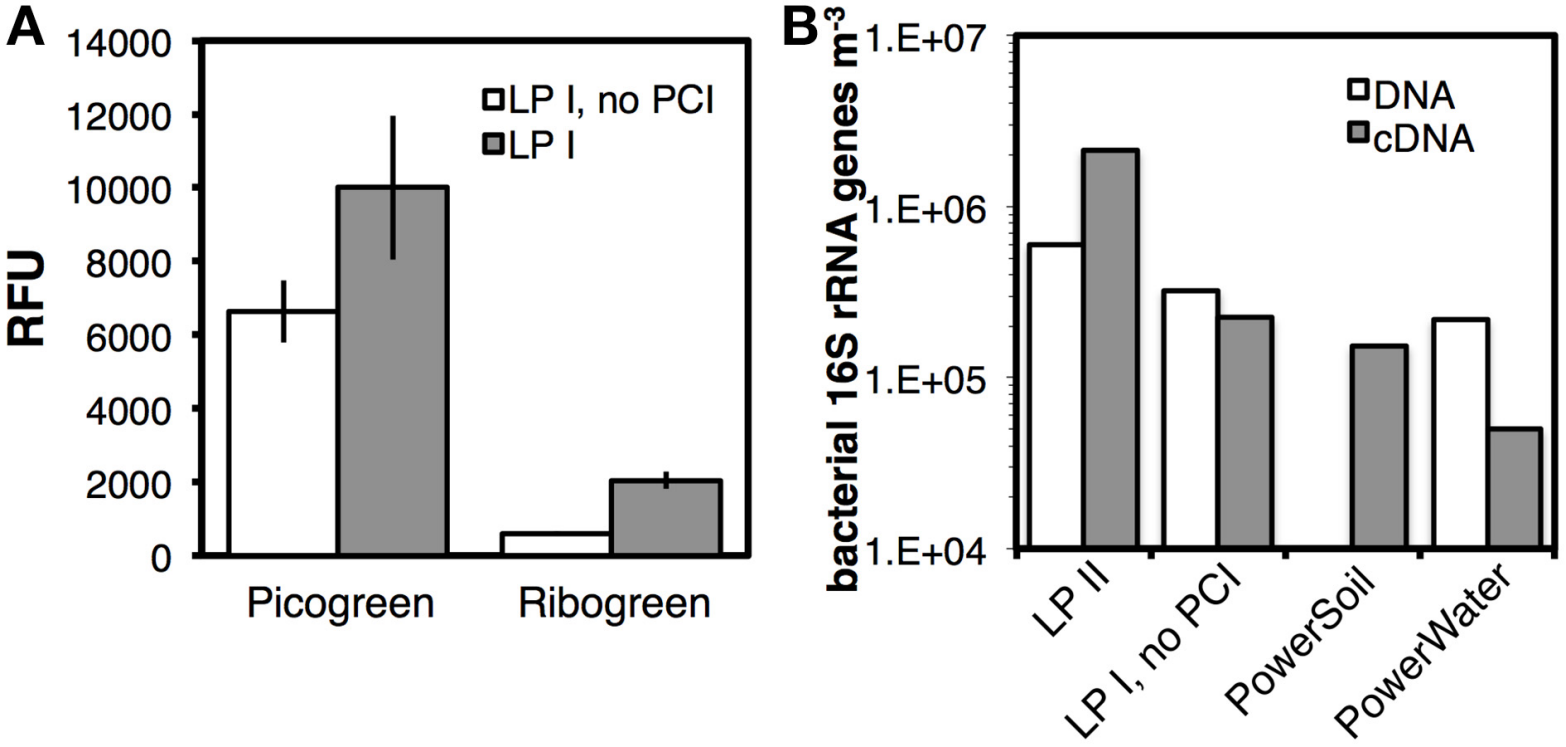
**(A)** DNA and RNA yields from surface sediment of Aarhus Bay Station M1, based on relative fluorescent units after DNA-staining with PicoGreen and RNA-staining with RiboGreen. DNA and RNA were extracted by a modification of LP I, in which only lysis solution I and PO_4_ but no PCI were added prior to bead-beating, and by LP I (same except with PCI). **(B)** DNA and cDNA yields from a lake water sample, treated by LP II, LPI omitting PCI, and two commercial kits by MO BIO Laboratories. Error bars in **(A)** indicate standard deviations of extractions that were run in triplicate.

We examined the compatibility of our protocol with RNA extraction. Using Aarhus Bay Station M1 surface sediment, we assessed whether LP I was effective at extracting RNA in addition to DNA, and compared RNA yields to a modification of LP I, in which PCI was omitted (Figure 9A). Fluorescence spectroscopic analyses indicated a ~50% higher DNA yield (*p* < 0.05) and a >200% higher RNA yield (*p* < 0.01) with the standard version of LP I.

We performed further tests, this time using lake water filtrates, and compared DNA and RNA yields following LP II and LP I without PCI, both without bead-beating (Figure 9B). While bacterial gene copy numbers doubled by including the freeze-thaw+heat incubation from LP II, cDNA copy numbers increased tenfold. We compared these data to bacterial copy numbers obtained with two commercial kits, the PowerWater Sterivex DNA Isolation Kit and RNA PowerSoil Total RNA Isolation Kit (adapted as described in the SOM). The PowerWater kit resulted in three times lower copy numbers than our method with LP II. Moreover, 16S rRNA gene copy numbers based on cDNA pools were 14 times lower with the PowerSoil kit, and 42 times lower with the PowerWater kit compared to those obtained with LP II.

After the simultaneous precipitation of DNA and RNA, additional purifications were necessary. Extracts were divided into two volumes, one for DNA and one for RNA purification. For DNA purification we used Protocol A of the Norgen CleanAll RNA/DNA kit. For RNA extraction, we compared Protocols C and D of the same kit, both of which are designed for RNA purification. We chose Protocol C due to the ~30% higher RNA recovery compared to Protocol D. After this first purification, RNA extracts were treated with DNAse to remove residual DNA. We tested possible variations of the manufacturers protocol, including longer digest times (2 h instead of 30 min) or addition of bovine serum albumin to improve the efficiency of the DNA digest, but found that neither resulted in improvements (Eickenbusch, 2014). Adding BSA increased the digestion of DNA, but also lowered RNA recovery by ~80%. After DNAse incubation, RNA extracts were purified one final time following Protocol C of the Norgen CleanAll RNA/DNA kit and were then ready for downstream analyses.

### Comparison of general protocol results to commercial protocols

**Figure 10:**
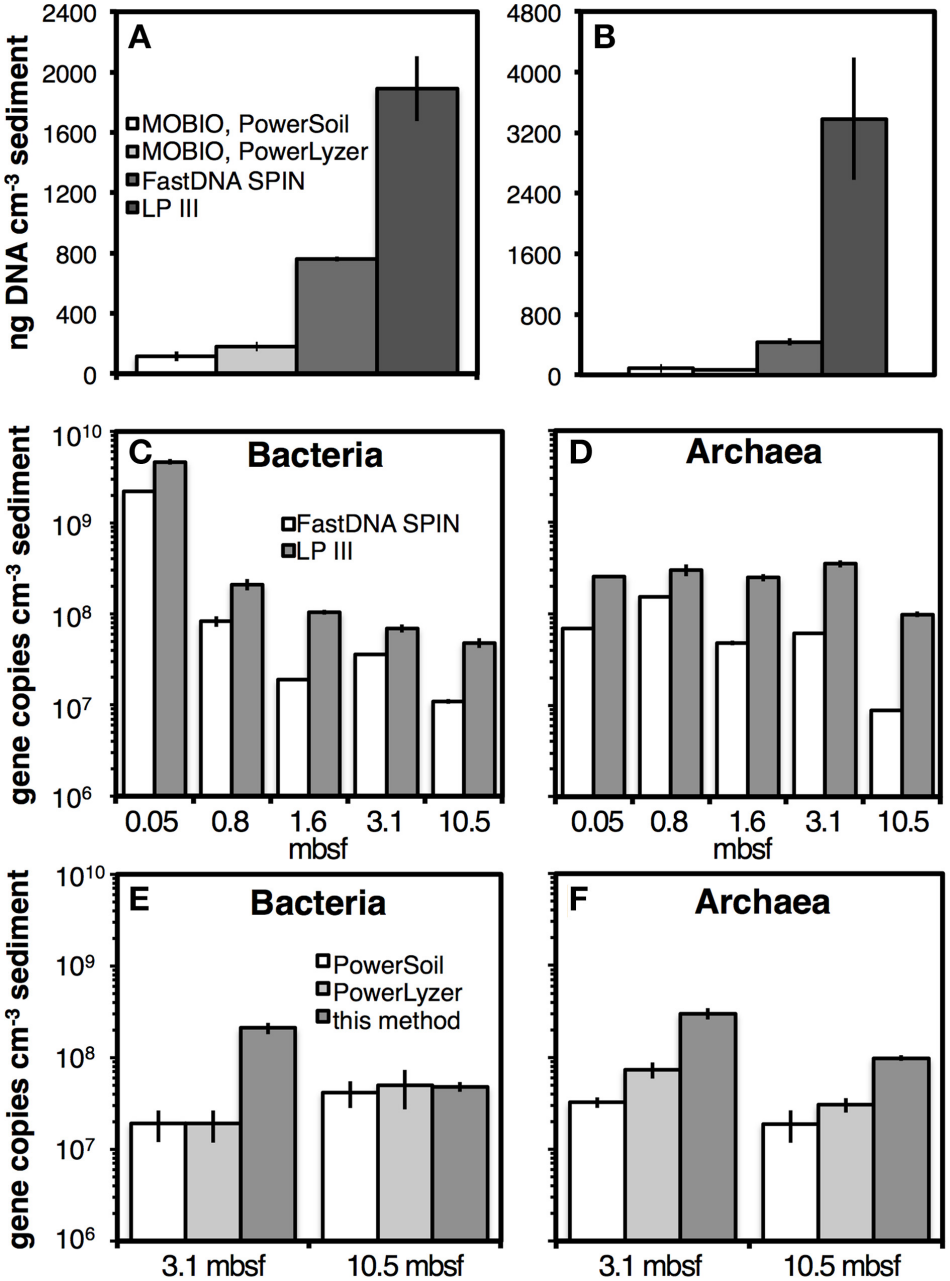
**DNA yield comparisons of our extraction method using LP III to commercial DNA extraction kits by MO BIO (PowerSoil, PowerLyzer) and/or MP Biomedicals (FastDNA SPIN)**. Quantifications were by fluorescence spectroscopy for Aarhus Bay Station M5 **(A)** and Aarhus Bay Station MIMOSA **(B)**. Quantifications were by qPCR of 16S rRNA genes on samples from Aarhus Bay Station M1 **(C–F)**. Five depths, from surface sediments to 10.5 mbsf were used for comparisons to the FastDNA SPIN kit **(C,D)**. Two samples were used for comparisons to both MO BIO kits **(E,F)**. Bead-beating was performed in extractions with the FastDNA SPIN kit and MO BIO PowerLyzer kit. Sediment samples from Station M5 and MIMOSA were both methanogenic and from 1.2 mbsf. Sediment samples from Station M1 were from 0.05, 0.8, 1.6, 3.1, and 10.5 mbsf, which corresponded to bioturbated surface sediment, sulfate reducing sediment, the sulfate-methane transition zone, methanogenic marine sediment, and a methanogenic soil layer, respectively. Error bars indicate standard deviations of extractions that were run in triplicate.

DNA yields obtained with this extraction protocol were significantly higher than those obtained with three widely used DNA extraction kits. Compared to the kits by MO BIO Laboratories, DNA yields were approximately one order of magnitude higher with this extraction protocol using LP III on samples from Aarhus Bay Stations M5 and MIMOSA (Figures 10A,B). Compared to the FastDNA SPIN kit, the difference was smaller, but our extraction protocol nonetheless yielded ~two to fivefold higher DNA yields (also Figures 10A,B). Using qPCR on DNA extracts from four marine sediment layers and one soil layer from Aarhus Bay Station M1, this method yielded 2–10 times higher bacterial and archaeal copy numbers than the FastDNA SPIN kit (Figures 10C,D). Using the two deepest samples from this station, similar differences in qPCR copy numbers were observed between this method and both MO BIO kits (Figures 10E,F). The only exceptions were bacterial copy numbers in the soil layer, which were nearly identical across all three methods (Figure 10E). The trends in qPCR results in Figures 10E,F were consistent with fluorescence spectroscopic measurements (Figure S8).

### Application of protocol to diverse environmental samples

Our protocol successfully extracted DNA from a wide range of samples, which ranged over 10 orders of magnitude in bacterial gene copy numbers and over 8 orders of magnitude in archaeal gene copy numbers (Table 4). The samples tested were from “extreme” environments, such as the deepest hole ever drilled by the IODP (C0020; Inagaki et al., 2012), the central part of the South Pacific Gyre (SPG 6), which is widely considered the “deadest” part of the world's oceans (D'Hondt et al., 2009, 2015), oligotrophic lacustrine clay sediment 800 m beneath the West Antarctic Ice Sheet (Subglacial Lake Whillans), and a petroleum-rich, shallow subsurface sediment layer with an *in situ* temperature of 93°C (Guaymas Basin, “Orange mat,” 0.31 mbsf). The highest bacterial gene copy numbers were found in coastal surface sediment from Aarhus Bay Station M1, whereas the lowest bacterial gene copy numbers were detected in the urban atmosphere in Aarhus. The highest archaeal gene copy numbers were found in oil-rich hydrothermal sediment of Guaymas Basin, whereas the lowest archaeal gene copy numbers were detected in deeply buried sediments of the Bering Sea and off Shimokita Peninsula. Our quantifications of sDNA and nsDNA pools indicate roughly equal proportions of both pools in coastal sediments of Aarhus Bay and shelf sediments off Namibia. In deep-sea sediments of the Bering Sea, sDNA pools exceed nsDNA pools sizes by factors of four to ten. RNA extracts were quantified in the two samples from Aarhus University, and our results indicate 3.5–12 times higher bacterial 16S cDNA copy numbers than gene copy numbers.

**Table 4:** **Nucleic acid yields from frozen environmental samples, measured by fluorescence spectroscopy or by qPCR**.

**Location**	**Site/Station**	**Fluor. spectroscopy Ng DNA cm^−3^**	**qPCR Bacteria**	**qPCR Archaea**	**Lysis Protocol (LP)**
Aarhus Bay	M1 (0.05 mbsf)	13070 ± 1330 (57/43)	4.6 ± 0.3 × 10^9^^*^ (42/58)	2.6 ± 0.0 × 10^8^^*^ (71/29)	LP III, metaPO_4_ (600 μmol g^−1^)
	M1 (0.80 mbsf)	1030 ± 91 (61/39)	2.1 ± 0.3 × 10^8^^*^ (51/49)	3.0 ± 0.4 × 10^8^^*^ (33/67)	“ “ “ “ “
	M1 (1.60 mbsf)	557 ± 21 (51/49)	1.0 ± 0.1 × 10^8^^*^ (43/57)	2.5 ± 0.2 × 10^8^^*^ (49/51)	“ “ “ “ “
	M1 (3.10 mbsf)	597 ± 39 (52/48)	6.9 ± 0.7 × 10^7^^*^ (50/50)	3.5 ± 0.3 × 10^8^^*^ (29/71)	“ “ “ “ “
	M1 (10.55 mbsf)	261 ± 34 (49/51)	4.8 ± 0.6 × 10^7^^*^ (48/52)	9.8 ± 0.7 × 10^7^^*^ (29/71)	“ “ “ “ “
Namibian Shelf	GeoB12806	ND	3.1 × 10^8^ (32/68)	4.7 × 10^7^ (46/54)	“ “ “ “ “
	GeoB12806	ND	1.9 × 10^7^ (56/44)	5.0 × 10^7^ (40/60)	“ “ “ “ “
Bering Sea	U1342B-1H-2	ND	4.0 × 10^7^	1.9 × 10^7^	LP II, dNTPs (300 μmol g^−1^)
	U1343E-80X-5	ND	2.5 × 10^5^ (83/17)	7.4 × 10^0^ (sDNA BD)	“ “ “ “ “
	U1344C-1H-3	ND	4.0 × 10^7^ (24/76)	1.3 × 10^7^ (19/81)	“ “ “ “ “
	U1344A-7H-2	ND	3.6 × 10^5^ (29/71)	1.5 × 10^5^ (10/90)	“ “ “ “ “
Guaymas Basin	“Orange mat”	ND	6.4 × 10^8^	3.5 × 10^9^	LP II, dNTPs (15 μmol g^−1^)
	“ “	ND	BD	1.7 × 10^4^	“ “ “ “ “
	“Yellow mat”	ND	3.9 × 10^8^	9.0 × 10^7^	“ “ “ “ “
Peru Trench	ODP 1230A-21-3	ND	1.0 × 10^5^ (56/44)	2.6 × 10^3^ (76/24)	LP III, metaPO_4_ (600 μmol g^−1^)
Off Shimokita Peninsula	C0020-10R-1	ND	2.0 × 10^3^	2.6 × 10^1^	LP II (2 × FT-heat), [metaPO_4_ (600 μmol g^−1^) + dNTPs (150 μmol g^−1^)]
	C0020-24R-3	ND	3.9 × 10^4^	BD	
	C0020 (165LMT)	ND	1.2 × 10^7^	7.4 × 10^1^	LP II (2 × FT-heat), dNTPs (15 μmol g^−1^)
	C0020 (61SMT)	ND	1.5 × 10^7^	9.9 × 10^3^	LP II (2 × FT-heat), dNTPs (150 μmol g^−1^)
	C0020 (61SMT)	ND	6.4 × 10^4^	5.0 × 10^2^	LP II (2 × FT-heat), dNTPs (150 μmol g^−1^)
Bornholm Basin	Station 024 7GC	ND	1.9 × 10^6^	4.6 × 10^5^	nsDNA, LP II (3 × FT-heat), [metaPO_4_ (600 μmol g^−1^) + dNTPs (150 μmol g^−1^)]
Subglacial Lake Whillans	Drill Site	ND	4.4 × 10^3^	ND	LP II, dNTPs (450 μmol g^−1^)
South Atlantic Gyre	GeoB12815	ND	5.5 × 10^8^	1.1 × 10^8^	LP II, dNTPs (300 μmol g^−1^)
South Pacific Gyre	SPG 1	ND	1.0 × 10^6^	1.4 × 10^5^	LP II (2 × FT-heat), [metaPO_4_ (600 μmol g^−1^) + dNTPs (30 μmol g^−1^)]
	SPG 6	ND	5.8 × 10^5^	6.7 × 10^4^	“ “ “ “ “ “ “ “ “ “ “
	SPG 6	ND	1.2 × 10^3^	4.6 × 10^1^	“ “ “ “ “ “ “ “ “ “ “
	U1371F-1H-1	ND	1.8 × 10^6^	2.0 × 10^4^	LP II, dNTPs (300 μmol g^−1^)
Juan de Fuca Ridge Flank	1362A-17R-3	ND	4.3 × 10^5^	8.0 × 10^2^	LP II, dNTPs (3000 μmol g^−1^)
Danish lake, north Jutland	N/A	ND	6.0 × 10^5^^*^, 2.1 × 10^6^^*^	ND	LP II, dNTPs (6 μmol L^−1^)
Greenland glacial lake	N/A	823	1.6 × 10^8^	4.1 × 10^6^	LP II, dNTPs (45 μmol L^−1^)
Aarhus University	2nd floor balcony	ND	6.8 × 10^2^^**^, 8.3 × 10^3^^**^	ND	LP II, dNTPs (0.006 μmol m^3^)

*Values in parentheses indicate percentages of sDNA and nsDNA (% sDNA/% nsDNA) for samples where DNA pools were separated. Italicized values under Bacteria are cDNA copy numbers. Samples were precipitated with ethanol-NaCl and purified by Protocol A of the CleanAll kit, except those from lake water and air, from which both DNA and RNA were extracted. The latter were precipitated with isopropanol-NaCl, DNA was purified by Protocol A, and RNA was purified by Protocol C of the CleanAll kit. ND, not determined; BD, below detection. Note that for sediment cores from Off Shimokita Peninsula (C0020-10R-1, C0020-24R-3) and Bornholm Basin sediment only nsDNA was quantified, despite separation of sDNA from nsDNA*.

* *Data is per cm^3^ instead of per gram*.

** *Data is per m^3^ instead of per gram*.

## Discussion

Using the modular nucleic acid extraction protocol, we successfully extracted nucleic acids from a wide range of environmental samples (Table 4). Adjustments to the protocol, such as inclusion of mechanical lysis by bead-beating, changes in the number of freeze-thaw+heat cycles, modifications in the PO_4_ dose, or incorporations of a second lysis treatment can all be made to maximize nucleic acid yields from a given sample. By adding two initial wash steps, sDNA pools can be separated from nsDNA pools. Without major adjustments, this protocol is also compatible with RNA extraction, thus enabling the efficient simultaneous extraction of DNA and RNA from the same sample. Higher DNA yields are obtained using this modular protocol compared to several commercial DNA and RNA extraction kits. This may be because the modular format allows the user to incorporate knowledge on specific sample characteristics and encourages initial tests before a decision on the final protocol permutation is made. In the following, we review the results of our tests and identify key variables for obtaining high nucleic acid yields. We then provide an outline of our final, modular method, and end with concluding remarks.

### Physical/mechanical lysis

#### Freeze-thawing+heating

The inclusion of lysis cycles consisting of freeze-thawing followed by 1-h incubations at 50°C, all under gentle agitation in the presence of chemical lysis solutions, increased DNA and RNA yields from many samples (Figures 3A,B,D, 9B). In one sample, increasing the number of cycles from one to three increased the DNA yields, while in others it lowered DNA yields (Figures 3E,F). Whether increases in DNA yields were due to freezing, heating, or longer chemical exposure to lysis reagents, due to combinations of or all three of these variables is not clear. Regardless, the combination of freezing, heating, and extended exposure to lysis chemicals proved effective at increasing yields of extracted DNA and RNA.

#### Bead-beating

Whether bead-beating increased nucleic acid yields depended on the sample type. In clay- and organic-rich sediments from Aarhus Bay, bead-beating prior to LP II yielded higher DNA yields than not bead-beating prior to LP II (Figures 1A,B). Though more tests are necessary for substantiation, increases in DNA yields from these sediments may occur because bead-beating breaks up clay structures and large organic particulates, and thereby enables more efficient penetration of lysis reagents and release of DNA from aggregates and organic debris. Similar DNA yield increases due to bead-beating were observed in organic-rich soils and sediments in a previous study (Miller et al., 1999). This benefit may be absent from sediments that are less cohesive and devoid of large organic particles or high organic matter content (Subglacial Lake Whillans; Figures 1C,D). In liquid samples (Figure 1E), or samples consisting of liquid mixed with silty sediment (Figure 1F), bead-beating even lowered gene copy numbers in extracts, possibly due to DNA shearing. Our results are in line with an earlier study (Lever, 2008), in which bead-beating increased DNA yields from cohesive clay-dominated lithologies, but decreased DNA yields from loose sand-dominated turbidites in subseafloor sediments of the Juan de Fuca Ridge Flank (U1301C). We do not question that direct mechanical cell lysis by bead-beating can contribute to higher lysis efficiency of difficult-to-crack cells, such as Gram-positive bacteria, bacterial endospores or fungal conidia (e.g., Zhou et al., 1996; Kuske et al., 1998; Wunderlin et al., 2013). Our results suggest, however, that the sample matrix also plays a role, and that the decision of whether to bead-beat with our method is best made based on pilot extraction tests rather than *a priori* assumptions.

### Chemical/enzymatic lysis

#### Enzymes

In the presence of high concentrations of SDS or guanidium hydrochloride+Triton X-100, we found no benefit of adding lysis-enhancing enzymes (Figures 2A,C). This suggests that the lysis chemicals added are at least as effective at lysing cells and releasing DNA from cells as the enzymes proteinase K, lipase, and lysozyme. We realize that this finding may not apply to all sample types. Past studies have demonstrated increased DNA yields due to enzymatic treatments (e.g., Krsek and Wellington, 1999; Hurt et al., 2001), and incubations using different kinds of enzymes could have increased DNA yields from our samples. Yet, in the absence of documented benefits of enzymes as part of our protocol, there is no justification for their use.

#### Detergent choice

Our tests also suggest that SDS does not increase DNA yields, if the main lysis buffer is guanidium-hydrochloride-based and amended with the detergent Triton X-100 (Figures 2B,D). On the contrary, Triton X-100 resulted in higher DNA yields than SDS addition, and the combination of SDS and Triton X-100 in some cases lowered DNA yields (Figure 2E, Figures S1A,B). Though we cannot explain the lowering of DNA yields by SDS, a possible explanation for the absence of increased DNA yields with SDS is that guanidium hydrochloride at the concentration used is an equally or more effective chaotropic agent than SDS. In addition, the non-ionic detergent Triton X-100 may more effectively bind and solubilize nonpolar components of cells, such as membrane lipids, than the ionic SDS. As a result, Triton X-100 may be a superior complement to guanidium hydrochloride in the lysis of cells and subsequent release of DNA.

#### CTAB+PVPP

Our results indicate that adding a second lysis solution containing CTAB and PVPP may increase DNA yields from high-biomass, organic-rich samples, but lowers DNA yields from low-biomass, organic-poor samples. A possible explanation for the lower DNA yields from low-biomass samples is the incomplete removal of CTAB during the PCI and/or CI purification steps. As CTAB binds strongly to DNA at NaCl concentrations <0.7 M (Murray and Thompson, 1980), and NaCl is efficiently removed during the DNA precipitation procedure, carryover of trace amounts of CTAB may result in effective precipitation and loss of DNA during the final elution step. This problem may be augmented in low-biomass, organic-poor samples, due to the absence of large amounts of polysaccharides to bind CTAB. Moreover, losses of DNA due to carryover of CTAB to the final extracts are likely to be most severe in samples containing low amounts of DNA to begin with.

#### Lysis protocol recommendation

Over the course of our tests, three lysis protocols evolved: LP I for extractions where speed was a high priority, i.e., high through-put applications or simultaneous extractions of DNA and RNA. Inclusion of PCI is necessary for a high nucleic acid yield using this protocol, but also bears the risk of phenol oxidation. LP III was developed for organic-rich samples containing high amounts of polyphenols and polysaccharides, which are PCR inhibitors and through complexation may inhibit the release of DNA during extraction. This protocol was the longest and the most labor-intensive, but worked well on organic-rich coastal sediments from Aarhus Bay. LP II is an intermediate between LP I and LP III and can be used across a wide range of samples—from organic-poor to organic-rich—often providing high yields of both DNA and RNA. Depending on the sample type and target molecule (DNA, RNA), LP II may work best with one, two, or three freeze-thaw+heat cycles. Reasons for the different extraction yields at different numbers of freeze-thaw+heat cycles are not clear. Some cells are more resistant and require longer lysis incubation times than others (Hoffman and Jarvis, 2003). In addition, there may be a dependency on the PO_4_ dose: if insufficient amounts of PO_4_ were added to prevent adsorption of DNA from lysed cells, then DNA concentrations in extracts may decrease over time.

### Adsorption prevention

#### pH

Our results underscore the critical importance of using an alkaline pH in DNA extraction solutions (Figures 2A, 4A, 8A,B), and indicate that using a pH of 9–10 instead of the widely used pH 8.0 of many conventional phosphate- or TE-based extraction buffers (e.g., Ogram et al., 1987; Zhou et al., 1996; Corinaldesi et al., 2005) results in higher DNA yields. This elevated pH does not denature DNA (Ageno et al., 1969) or induce cell lysis on the samples tested (Figures 8C,D), and is thus compatible with the separate extraction of nsDNA and sDNA. Our results are consistent with previously documented reductions in DNA adsorption to organic and inorganic clays and nanoparticles, when the pH was raised above 8 (Cai et al., 2006; Tanaka et al., 2009). Our results indicate that a pH of >9 is especially important in maximizing sDNA yields. Due to a pK_a_ of 1–3 (Brown et al., 2010), virtually all phosphodiester groups of DNA are deprotonated in the pH range examined (pH 5–10), and thus not significantly affected by raising the pH above 8. Instead, if experiments with silica or aminosilane-modified magnetic nanoparticles provide an indication, then a combination of electrostatic and hydrophobic factors might be at play. These could cause electrostatic repulsion of DNA from negatively charged solid surfaces at high pH, and increase cation bonding and hydrophobic interactions of DNA with solid surfaces at low pH (Isailovic et al., 2007; Geng et al., 2009).

#### Phosphate

Consistent with past studies (e.g., Ogram et al., 1987; Holben et al., 1988; Pietramellara et al., 2001; Tanaka et al., 2009; Direito et al., 2012), our data indicate that pyrophosphate, hexametaphosphate, and dNTPs are all effective at reducing DNA adsorption and even desorbing DNA (Figures 4C,D, 8A,B). We detect no significant difference in the efficacy of pyrophosphate and hexametaphosphate in preventing adsorption (Figure S3A), and also had excellent results using dNTPs (Table 4). Future studies may further compare the efficacies of these three PO_4_ sources in preventing adsorption and releasing DNA from different sample types.

In addition to yield increases, we observed diminished DNA yields due to high PO_4_ additions in certain samples (Figure 4E). These decreases occurred at PO_4_ additions that were lower than those resulting in the highest DNA yields on other samples, indicating that the optimal amount of PO_4_ added depends on the sample type. A possible explanation is that samples with a high DNA adsorption capacity, require higher PO_4_ doses to release DNA into aqueous solution than samples with lower DNA adsorption capacity. Since the DNA adsorption capacity of any sample type has a limit, PO_4_ doses exceeding this capacity are carried over to the precipitation step. We observed that in samples with excess PO_4_, DNA pellets were not only larger, but sometimes had a darker color, suggesting that enhanced PO_4_ carryover can also increase the carryover of non-nucleic acid organic matter, e.g., humic compounds. Without influencing the yield, enhanced PO_4_ and/or organic matter carryover then increased PCR inhibition in the absence of post-extraction purification (Figure S4C). Moreover, during post-extraction purification using the Norgen CleanAll RNA/DNA Kit, this carryover increased DNA loss. Presence of high amounts of PO_4_ might have increased DNA loss to washout prior to elution due to competitive binding of PO_4_ or co-extracted humic substances to silica membranes. In addition, carryover of PO_4_ and co-extracted organic matter might have reduced dissolution efficiency of DNA and resulted in DNA not passing the silica membrane during elution. Substantial losses during nucleic acid purification by silica columns were previously documented by Lloyd et al. (2010) and attributed to competitive binding by co-extracted humic acids to silica membranes. The fact that a particularly high DNA loss occurred at high PO_4_ doses applied to drilling mud on the *D/V Chikyu* (compare Figure 4E to Figure 4B), which does not include humic acids as a typical ingredient (Masui et al., 2008; Yanagawa et al., 2013), however, suggests that PO_4_ carryover is also a part of the problem (also see “Precipitation” Section in Discussion).

### Purification

Our tests with phenol, chloroform, and isoamylalcohol in different combinations showed a clear positive effect of isoamylalcohol in stabilizing interfaces of aqueous and organic phases. Inclusion of isoamylalcohol with chloroform or phenol-chloroform enabled cleaner transfers of aqueous supernatants and often lowered the number of necessary CI or PCI washes. DNA washes with PCI and CI or only CI were essential for obtaining high DNA yields, possibly due to a combination of factors, including the removal of detergent and other bipolar and apolar compounds (Figures 5A,C,D). Yields after one PCI wash followed by one or two CI washes were typically equivalent to DNA yields after 2–3 CI washes (Figure 5B). PCI was more effective at precipitating undesired compounds at the aqueous-organic interface, but became oxidized when used with many samples and was thus potentially damaging to DNA. Adding reductants such as 2-hydroxyquinoline, β-mercaptoethanol, or TCEP did not solve this problem. Except on samples where phenol oxidation is absent and carryover of undesired compounds is not effectively prevented by CI washes, we thus recommend only using CI for the purification prior to precipitation.

### Precipitation

Our precipitation tests indicate that the best precipitation method depends on the goals of the study. DNA precipitation efficiency is highest using ethanol-NaCl or PEG-NaCl, which can both provide virtually full DNA recovery (Figure 6, Figures S4C–E). A similarly high recovery is attained with a solution combining ethanol with PEG and NaCl; however, this solution has no apparent advantages over precipitation with ethanol-NaCl or PEG-NaCl. Isopropanol-based extraction solutions have slightly lower DNA yields, but are also suited for RNA precipitation.

In precipitations with PEG-NaCl, the choice of PEG (6000 or 8000) does not influence the DNA yield. However, great care must be taken to not pipet off the frequently invisible DNA pellet (see Section “Precipitation” in Results), and autoclaving of PEG solution should also be avoided (Figure S4D). Possibly autoclaving induces hydrolysis of PEG polymers and shorter PEG polymer fragments have a lower DNA precipitation efficiency. In addition, PEG precipitation has clear advantages over ethanol-NaCl precipitation: DNA pellets are much smaller, because the solubility of PO_4_ is considerably higher in 30% PEG-1.6 M NaCl solution than in ethanol-NaCl solution (~70% ethanol, ~0.5 M NaCl). As a result, purifications of DNA pellets using post-extraction cleanup kits are often not necessary.

In addition to saving time and reducing expenditures, omitting post-extraction cleanups has a second major advantage: excess PO_4_ carryover in sDNA extracts induces selective loss of short DNA fragments during cleanup with the CleanAll kit (Figure S7). These short sDNA fragments might, however, be of particular interest to certain fields, e.g., research on fossil DNA pools (Willerslev and Cooper, 2005; Corinaldesi et al., 2011; Coolen et al., 2013). The selective bias against short DNA fragments was not seen when 100-bp DNA ladder was precipitated with ethanol-NaCl or purified using the Norgen CleanAll RNA/DNA kit in the absence of added PO_4_ (data not shown). This not only confirms our earlier interpretation that high PO_4_ carryover, and possibly PO_4_-induced carryover of humic acids, results in DNA loss during cleanup on silica columns. It also indicates that PO_4_ carryover onto silica columns induces particularly high DNA losses in small DNA size fractions. The reason why this trend was only seen in sDNA extracts is likely that, during DNA desorption in the second hour of sDNA extraction, most PO_4_ ends up in the sDNA extract. The solution to this problem is to perform PEG precipitation on sDNA extracts (Figure S7). If necessary, PEG precipitation can be followed by purification with the CleanAll kit, which works well after PEG precipitation due to the low PO_4_ carryover. Reducing the PO_4_ dose to levels that result in less PO_4_ carryover into final sDNA extracts might also work, as long as it does not increase sDNA and nsDNA to adsorption.

### Final purification

The Norgen CleanAll RNA/DNA kit proved easy-to-use, versatile in its effective purification of both DNA and RNA, and had excellent nucleic acid recovery except in cases with high PO_4_ carryover (Figure 7). Final extracts after purification were typically devoid of visible coloration, were suitable for spectrofluorometric quantification on the ND-3300, an instrument that is highly sensitive to impurities from detergent or humic substances, and showed no signs of PCR inhibition. High purity and absence of PCR inhibition is especially paramount in PCR-based applications on low-biomass samples, where dilutions of nucleic acids can easily lower DNA or RNA concentrations below background contamination.

### Separation of DNA pools

Our tests indicate that the separation of sDNA and nsDNA pools is feasible. To effectively extract sDNA, high pH and high PO_4_ concentrations in extraction solutions are critical. An extraction buffer with pH 8 or low PO_4_ concentrations only recovers a small fraction of the sDNA that is extracted at pH > 9 and high PO_4_ (Figure 8A). By contrast, a pH of 8 and low PO_4_ dose recovers ~70% of the nsDNA that is extracted at high pH and high PO_4_ (Figure 8B). The observed trends allude to differences in origin between sDNA and nsDNA pools. sDNA may primarily consist of DNA that is adsorbed or weakly bound by intermolecular forces. Using an ample supply of PO_4_, which competes with sDNA for adsorptive surfaces, and an alkaline pH, sDNA readily dissolves. By contrast, the nsDNA pool may be tightly complexed within living cells, viruses, or non-soluble organic matter. The latter may include detrital DNA-binding proteins and humic substances (Nielsen et al., 2006). Part of the nsDNA pool may also be locked within clay aggregates or dead cells. This nsDNA is only released into solution after cell membranes, cell walls, DNA-binding proteins, and other structures have been dismantled by physical and/or chemical lysis agents.

Inevitably, our interpretations concerning characteristics of sDNA and nsDNA pools in sediments require further confirmation. Yet, the results of additional tests are in line with our interpretations. For instance, we can rule out a large contribution of living cells to sDNA pools, based on the absence of cell lysis during sDNA extraction (Figure 8D), and because nearly 100% of the extracted sDNA readily passes through 0.2-μm pores (Figure S5A). It also appears that—at least in marine sediment—the bulk of sDNA is non-viral, as it passes through 0.02-μm pore sizes (Figures S5A–C). By contrast, in a deeply buried terrestrial soil layer, most sDNA was within the 0.02–0.2 μm size fraction, possibly indicating a higher viral contribution. Alternatively, more sDNA might have been complexed with dissolved organic matter in the 0.02–0.2 μm size fraction.

Both sDNA and nsDNA are measurable by fluorescence spectroscopy and PCR-amplifiable using bacterial and archaeal 16S rRNA gene primers (Table 4). The relative contributions of sDNA and nsDNA appear to vary considerably, with sDNA accounting for 10–83% of total DNA based on qPCR results. Fluorescence spectroscopic and qPCR results compare well, though we on average observed a higher fraction of sDNA according to fluorescence spectroscopic measurements. This could be due to a higher non-amplifiable DNA fraction in the sDNA pool compared to the nsDNA pool.

### Comparison to commercial protocols

Higher DNA yields were achieved with this modular protocol than with several widely used commercial kits for the extraction of DNA and/or RNA from soil and filtrates. Given that the active ingredients of various commercial protocols are proprietary, it is not possible to identify why this modular protocol yielded higher DNA or RNA yields. We attribute the higher yields at least in part to the fact that we took into account specific sample characteristics and, in many cases, performed initial extraction trials in which variables, such as bead-beading or PO_4_ dose were tested, before deciding on a final extraction protocol.

### Final protocol

**Figure 11:**
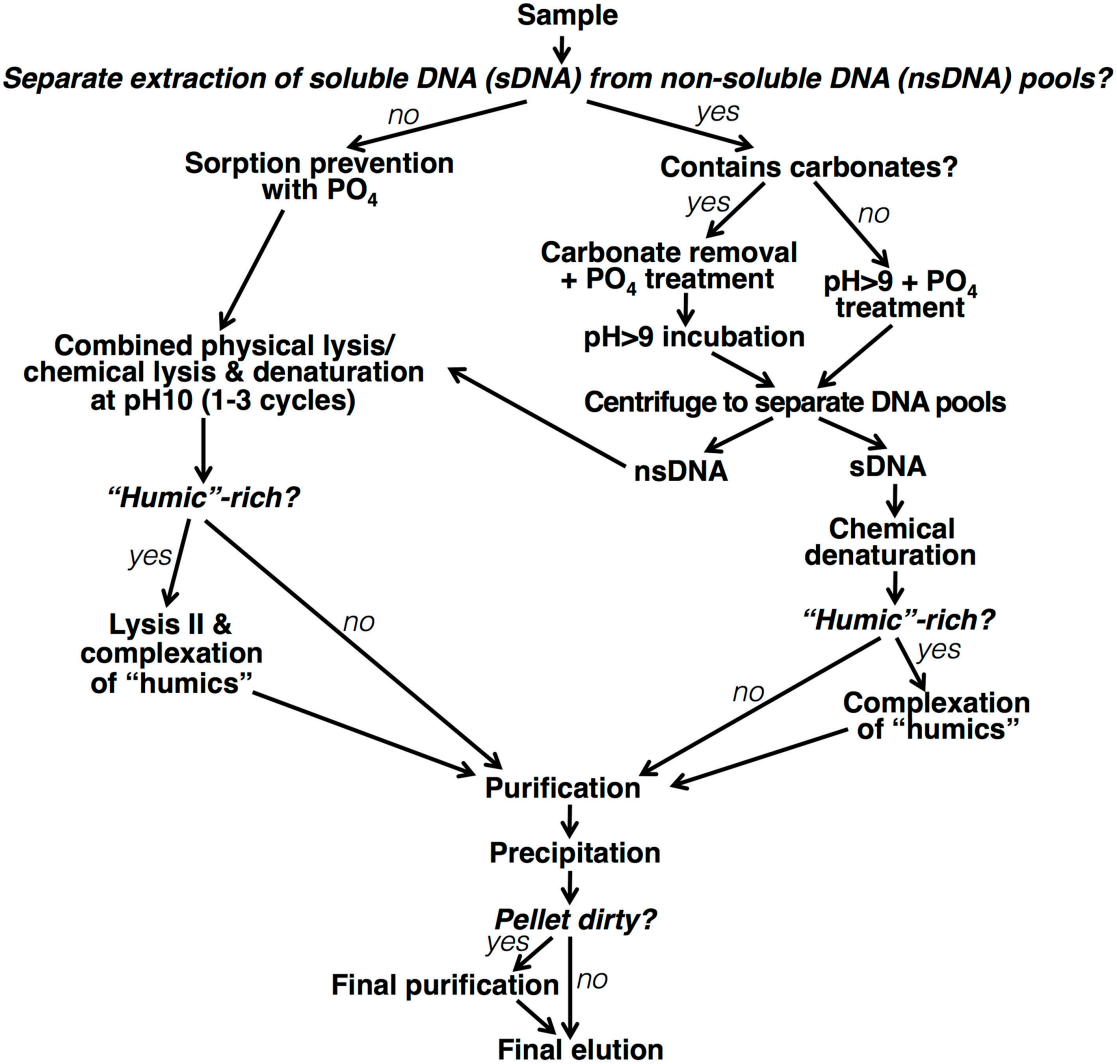
**Flow chart of modular nucleic acid extraction protocol**.

A flow chart of our final modular DNA/RNA extraction protocol is presented in Figure 11. In the following, we provide a written outline of the protocol and its various permutations. All recipes for reagents can be found in the SOM.

#### Weighing and aliquotting of sample

The desired amount of sample is placed into a pre-weighed centrifuge tube. If bead-beating is performed in later steps, screw-cap centrifuge tubes are used, which are filled to ~15% of the total volume with 0.1-mm zirconium silicate beads prior to sample addition.

#### PO_4_ addition

Concentrated PO_4_ solution is added to prevent adsorptive losses of nucleic acids. The optimal PO_4_ dose depends on the adsorption capacity of the sample, and can only be determined empirically. For total DNA extractions from organic-rich and sandy sediments, 10–100 μmol PO_4_ g^−1^ are often sufficient, whereas for organic-poor clay sediments with high sorption capacity, 100–1000 μmol PO_4_ g^−1^ might be necessary. Water and air samples that are filtered onto membranes may only require 10 μmol L^−1^ of water or air, or less. If nsDNA and sDNA are extracted separately, then higher PO_4_ additions, e.g., 100–1000 μmol PO_4_ g^−1^ for organic-rich sediment samples, may be necessary to maximize desorption of sDNA. After addition of PO_4_ solutions, samples are gently mixed to coat the entire sample with PO_4_ solution. If RNA extraction is intended, samples should be kept on ice throughout the PO_4_ addition procedure.

#### Separation of sDNA and nsDNA

This step is skipped unless the separate extraction of sDNA and nsDNA pools is desired. There are two options for the separate extraction of sDNA and nsDNA. For carbonate-rich samples, 1 volume CDM is added per equivalent weight of sample, e.g., 0.2 mL CDM per 0.2 g of sediment. Samples are then gently mixed at room temperature for 1 h, e.g., using a shaker incubator, rotator mixer, or hybridization incubator with a rotisserie assembly. Afterward, 8 volumes of 10× TE buffer are added, e.g., 1.6 mL 10× TE per 0.2 g of sample. Samples are then gently mixed at room temperature for a second hour. For samples containing low amounts of carbonate, 9 volumes of 1× TE buffer are simply added, e.g., 1.8 mL 1× TE per 0.2 g, and samples are gently shaken for 2 h at room temperature. We have seen no detrimental effect of applying the protocol for carbonate-rich samples to carbonate-poor samples and—when in question—recommend this protocol.

After 2 h, samples are centrifuged for 20 min at 10,000×g. The supernatant containing sDNA is filtered through a 0.2-μm filter and transferred to a clean tube. Further processing of sDNA is discussed at the end of this protocol. For extraction of nsDNA the following step is used.

#### Cell lysis

The first step of the cell lysis procedure is the addition of 2.5 volumes lysis solution I, e.g., 500 μL lysis solution I per 0.2 g of original sample. Samples are gently mixed to ensure homogenization and then frozen at −80°C. Afterward, our modular protocol offers three separate lysis protocols (LPs I–III), with the best protocol depending on the sample type and scientific goal.

#### LP I

This short combined mechanical and chemical lysis procedure is suited for simultaneous extraction of DNA and RNA and high-throughput applications. 2.5 volumes of cold PCI are added to the frozen samples already containing PO_4_ solution and lysis solution I, e.g., 500 μL PCI per 0.2 g of the original sample. Samples are allowed to thaw. After thawing, samples are vortexed for 10 s, and then bead-beaten at medium to high speed for 30 s to 1 min, with the duration of bead-beating representing a compromise between cell lysis efficiency and nucleic acid shearing. Using a TissueLyzer LT 2500 (Qiagen) or FastPrep-24 (MP Biomedicals) we have had good results from cohesive, clay-rich samples using 1 min at maximum speed. For loose samples or coarse-grained samples, lower speeds may be preferable, and vortexing for 30 s might be a superior alternative.

#### LP II

This protocol is perhaps the most versatile of the three LPs, providing high DNA and RNA yields from a wide range of samples, including ultra-oligotrophic sediment and basalt samples. Samples amended with PO_4_ solution and lysis solution I are thawed, homogenized by vortexing for 10 s, and incubated for 1 h at 50°C, e.g., using a shaker incubator, rotator mixer, or hybridization incubator with a rotisserie assembly. Bead-beating for 30 s to 1 min, or vortexing horizontally attached screw-cap tubes with beads for 10 min, may substitute for brief 30 s vortexing prior to the incubation, but is often not necessary. Up to two additional freeze-thaw+heat cycles may increase yields on certain samples.

#### LP III

The lengthiest of the three protocols is perhaps the most suitable for DNA extractions from organic-rich samples containing high amounts of polysaccharides, humic substances, and (recalcitrant) cell structural components. Samples amended with PO_4_ solution and lysis solution I are thawed, vortexed for 10 s at high speed, and incubated for 1 h at 50°C, e.g., using a shaker incubator, rotator mixer, or hybridization incubator with a rotisserie assembly. After the following, second freeze-thaw+heat cycle, 2.5 volumes lysis solution II, e.g., 500 μL lysis solution II per 0.2 g of original sample, are added, and two more freeze-thaw+heat cycles are performed. During the final 1-h incubation, the temperature is raised to 65°C to promote binding of CTAB to organic components.

After bead-beating in LP I, and after the (final) 1-h incubations in LPs II and III, samples are centrifuged for 10–20 min at 10,000×g and 4°C, and supernatants containing nucleic acid extracts are transferred to clean centrifuge tubes. The necessary centrifugation time depends on the temperature and volume of the sample prior to centrifugation. After the 10–20 min centrifugation period, all samples should be ~4°C cold. With samples treated by LP I, care is taken to transfer as much aqueous supernatant as possible, while avoiding the organic phase or precipitates at the aqueous-organic interface. After centrifugation of samples treated with LPs II and III, as much supernatant is transferred as possible, while keeping transfer of sediment particles to a minimum.

#### Purification

To remove detergents and residual bipolar organic compounds, supernatants are washed with 1 volume of CI. After CI addition, aqueous supernatants are emulsified with CI by vortexing at maximum speed for 10 s, or until thoroughly mixed. After centrifugation for 10 min at 10,000×g and 4°C, tubes are kept cold, and—avoiding the CI phase and precipitates at the organic-aqueous phase—aqueous supernatants are transferred to clean tubes. The whole procedure consisting of CI addition, vortexing, and centrifugation is repeated one more time. During the second transfer of supernatant there should be no carryover of CI or visible precipitates. With certain, very “clean” samples, the second CI transfer can be skipped, while with other samples, a third CI wash might be necessary. For samples that are very rich in humic substances, PCI may be used instead of CI in the first wash step, though only if strong phenol oxidation, indicated by conspicuous reddish discoloration after vortexing, is absent.

#### Precipitation

For DNA work, we recommend Ethanol-NaCl or PEG-NaCl precipitation. Ethanol-NaCl precipitation is easier to perform than precipitation with PEG-NaCl, due to formation of a clear, solid pellet after centrifugation. However, ethanol-NaCl precipitation more often than PEG-NaCl precipitation requires additional purification to prevent PCR inhibition or enable spectrofluorometric quantification. At high PO_4_ carryover, this additional purification can be associated with significant DNA losses. For the simultaneous extraction of DNA and RNA, or only RNA, both isopropanol-NaCl and isopropanol-ammonium acetate precipitations are suitable.

The first step in every precipitation protocol is the addition of linear polyacrylamide (LPA) to purified supernatant to a final LPA concentration of 20 μL mL^−1^. LPA is a co-precipitant, which significantly enhances the precipitation efficiency of nucleic acids (Bartram et al., 2009). LPA is fully dissolved and homogenized with the sample. For Ethanol-NaCl precipitations, samples are then homogenized with 0.2 volumes 5 M NaCl solution, and subsequently with 2.5 volumes of pure ethanol. For isopropanol-NaCl precipitations, samples are homogenized with 0.1 volumes of 5 M NaCl solution followed by 1.5 volumes of isopropanol. For isopropanol-ammonium acetate precipitations, samples are mixed with 0.5 volumes of 7.5 M ammonium acetate solution, followed by 1.5 volumes of isopropanol. For PEG precipitations, samples are simply mixed with a non-autoclaved solution containing 30% *w/v* PEG 6000 or PEG 8000 and 1.6 M NaCl. DNA-targeted Ethanol-NaCl and PEG-NaCl precipitations then proceed through incubation in the dark at room temperature for 2 h. These precipitations can also be done overnight, but should be refrigerated at 4°C in this case. RNA- and RNA+DNA-targeted isopropanol precipitations proceed in the dark at −20°C for a minimum of 2 h and can also be stored overnight.

After the incubation period, samples are centrifuged at 14,000×g for 30 min. For tubes containing large fluid volumes, e.g., half-full to full 15-mL or 50-mL tubes, the centrifugation period is increased to 45 or 60 min. The centrifugation temperature is room temperature for DNA applications, and +4°C for downstream applications involving RNA. The supernatant is carefully decanted or pipetted off (never decant after PEG precipitations).

After centrifugation following Ethanol-NaCl precipitation, the pellet containing nucleic acids is air-dried. Prior to drying, 1–2 washes with 70% ethanol may be included. If further purification with the Clean All kit is planned, these washes, which effectively remove NaCl, but not PO_4_, are not necessary. An efficient way of drying nucleic acid pellets, while minimizing contamination risk, is to use a vacuum centrifuge set to 40–50°C. To remove isopropanol, pellets precipitated by isopropanol-containing solution are washed once with 70% ethanol, centrifuged for 10 min at 14,000×g, and air-dried after removal of the supernatant. To remove PEG from pellets that have been precipitated with PEG-NaCl, pellets are washed twice with 70% ethanol followed by 10 min centrifugation at 14,000×g. Two washes are necessary, as removal of PEG solution has to be done with great care to avoid pellet loss, and applying this extra care results in higher PEG carryover between washes.

All pellets are dissolved in 100–200 μL of water and are then ready for final purification, e.g., using the Norgen Clean All RNA/DNA kit. PEG precipitated DNA is ready for downstream quantifications and PCR-based applications without further cleanup.

#### Cleanup of sDNA

After sDNA extraction (see “Separate extraction of sDNA and nsDNA”), the sDNA is concentrated by Ethanol-NaCl or PEG-NaCl precipitation. The pellet is dissolved in 100 μL water, amended with 500 μL of lysis solution I, and incubated for 1 h at 50°C under gentle shaking, e.g., using a shaker incubator, rotator mixer, or hybridization incubator with a rotisserie assembly. With organic-rich samples, 500 μL of lysis solution II may be added afterward, and a second 1-h incubation at 65°C may be performed.

After the (final) 1-h incubation, samples are centrifuged for 10–20 min at 10,000×g and 4°C, and supernatants containing nucleic acid extracts are transferred to clean centrifuge tubes. From hereon the regular protocol is resumed from the “Purification” section.

## Conclusions

We have developed a modular method for the extraction of DNA from a wide range of environmental sample types. Through minor adjustments in the protocol, which likely address differences in sample matrices, mineralogies, organic matter contents, organic matter compositions, and phylogenetic community compositions, DNA yields from different sample types are substantially increased. The fact that different variations of our protocol work best for different sample types is not unique to our protocol, but a general feature of nucleic acid extraction protocols (e.g., Sørensen et al., 2004). This does not mean that this DNA extraction protocols needs to be fine-tuned and optimized for every individual sample in a study. It is, however, advisable to perform pilot extraction tests at the beginning of every study involving new sample material, in which samples tested represent the range of sample types studied. Key variables such as the suitable PO_4_ dose, lysis protocol, and precipitation method are then evaluated for each sample type. Moreover, in studies where standardized lysis protocols are necessary, it is possible to design a lysis module that works across all samples. If desired, this lysis module can then be combined with other standardized modules, or with modules that are optimized for non-lysis-related variables affecting nucleic acid recovery, such as PO_4_ dose.

In addition to enabling extraction of DNA from a wide range of sample types, this protocol enables the simultaneous extraction of RNA, and the separate extraction of sDNA and nsDNA. A clear advantage of using the same protocol for DNA and RNA extraction is that it provides insights to the phylogenetic compositions of active and less active community members without phylogenetic biases induced by separate extractions or extraction methods. The fact that different DNA pools can be analyzed on the same samples, moreover, invites further investigations on the nature of these two pools with respect to their phylogenetic origin, turnover, and preservation. These investigations will be crucial to assessing the extent to which molecular biological assays on total DNA extracts accurately inform on living microbial communities in environmental samples.

### Conflict of interest statement

The authors declare that the research was conducted in the absence of any commercial or financial relationships that could be construed as a potential conflict of interest.

## Supplementary material

Figure S1 **(A,B)** 16S rRNA gene copy numbers cm^−3^ sediment corresponding to the DNA extracts shown in (Figure 2D (**A**: Bacteria; **B**: Archaea). **(C)** DNA yields obtained after incubating sediments from Aarhus Bay Station M5 with a second lysis solution containing two concentrations of CTAB (2%, 0.5%) and PVPP (0%, 0.1%). Error bars indicate standard deviations of triplicate DNA extractions.

Figure S2 **(A)** Comparison of bacterial and archaeal 16S rRNA gene copy numbers after DNA extraction by LP I compared to LP I without PCI. Surface sediment from Aarhus Bay Station M1 was used as test material. Error bars indicate standard deviations of triplicate DNA extractions. **(B,C)** Comparison of bacterial **(B)** and archaeal **(C)** 16S rRNA gene copy numbers after DNA extraction by LP II compared to LP III from two oligotrophic sediments [Bering Sea, South Atlantic Gyre (SAG)] and subseafloor basalt (Juan de Fuca Ridge Flank).

Figure S3 **(A)** Effect of adding the same concentration of pyrophosphate and hexametaphosphate on DNA yields from subsurface sediments of Aarhus Bay Station M5 and M1. Concentrations of pyrophosphate and hexametaphosphate correspond to 80 and 240 μmol PO_4_ g^−1^ sediment, respectively. **(B)** Bacterial and archaeal 16S rRNA gene copy numbers in DNA extracts from a Greenland glacial lake after two different amounts of PO_4_ had been added in the form of dNTPs (5 and 15 μmol dNTPs g^−1^). **(C)** Bacterial and archaeal 16S rRNA gene copy numbers in DNA extracts from Subglacial Lake Whillans. The PO_4_ treatments included 450 μmol PO_4_ g^−1^ as dNTPs (“450”), small amounts of dsDNA (“dsDNA”; 0.001 μmol PO_4_ g^−1^), and 450 μmol PO_4_ g^−1^ as dNTPs plus 0.001 μmol PO_4_ as dsDNA (“450+dsDNA”). Error bars indicate standard deviations of triplicate extractions **(A)**, or ranges of duplicate extractions **(B,C)**.

Figure S4 **Comparison of DNA yields, expressed in ng DNA or gene copy numbers, using different precipitation methods. (A)** DNA yields by Ethanol-NaCl precipitation at room temperature and at −20°C using DNA extracts from Aarhus Bay Stations M5 (1.2 mbsf) and M1 (3.1 cmbsf). **(B)** DNA yields by Ethanol-NaCl precipitation after LP III. The first treatment with ~1.2 M NaCl left from Lysis Solution II prior to ethanol addition, the second treatment with addition of 0.2 volumes of 5 M NaCl, which raised the NaCl concentration to ~1.8 M prior to ethanol addition. **(C)** Effect of precipitation method at two different dNTP additions on PCR inhibition based on quantifications of bacterial 16S rRNA gene copy numbers in original and tenfold diluted extracts. DNA was extracted from Aarhus Bay Station M1 surface sediment and PCR-amplified after precipitation, without further purification by the Norgen CleanAll kit. I–III: 15 μmol PO_4_ added g^−1^ sediment, IV–VI: 150 μmol PO_4_ g^−1^ sediment. Precipitation methods: I, IV: PEG-NaCl; II, V: PEG-ethanol-NaCl; III, VI: EtOH-NaCl. **(D)** Effect of autoclaving PEG-NaCl solution on DNA recovery from three dilutions of 100-bp ladder (original concentration: 500 ng DNA μL^−1^). **(E)** Effects of centrifugation force, MgCl_2_ addition, or manipulating the pH of the PEG 8000-NaCl solution with acetate buffers on DNA recovery by PEG 8000-NaCl precipitation. Aliquots of the same homogenized DNA extract from Aarhus Bay Station M5 were diluted tenfold and used for these tests. I: conventional PEG precipitation with 2 volumes of solution containing 30% PEG 6000-1.6 M NaCl (pH 8.3); II: as I, but amending PEG-NaCl solution with 0.1 volumes of 300 mM MgCl_2_ (pH 8.4); III: as I, but 0.1 volumes of 3 M Na acetate added to PEG solution (pH 8.7); IV: as I, but 0.1 volumes of 1:1 (*v:v*) 3 M acetic acid: 3 M Na acetate added (pH 4.6); V: as I, but 0.1 volumes of 9:1 (*v:v*) 3 M acetic acid: 3 M Na acetate added (pH 3.7); VI: as I, but 0.1 volumes of 99:1 (*v:v)* 3 M acetic acid: 3 M Na acetate added (pH 3.2); VII: as I, but 0.1 volumes of 3 M acetic acid added (pH 3.1); No ppt, control consisting of non-precipitated original extract. Error bars in **(A–C)** indicate standard deviations of triplicate extractions, error bars in **(D)** indicate data ranges of duplicated precipitation tests.

Figure S5 **(A)** Amount of sDNA that passes through a 0.2-μm filter compared to total sDNA. Samples were from Aarhus Bay Stations M5 and MIMOSA. sDNA was extracted by the same method as in (Figure 8C. **(B)** Amount of sDNA in the 0.02–0.2 μm fraction compared to the amount of sDNA in the <0.02 μm fraction. Data from five different depths at Aarhus Bay Station M1, of which the uppermost depths (0.05 mbsf, 0.80 mbsf, 1.40 mbsf, 3.10 mbsf) correspond to marine sediment, and the lowermost depth (10.5 mbsf) corresponds to a soil layer. **(C,D)** Bacterial **(C)** and archaeal **(D)** qPCR results on the same DNA fractions and samples as shown in **(B)**. Error bars indicate standard deviations of triplicate extractions.

Figure S6 **Effect of freezing on nsDNA yields from Aarhus Bay Station M5**. Error bars indicate standard deviations of triplicate extractions.

Figure S7 **DNA size distributions after nsDNA and sDNA extract precipitation by PEG 8000-NaCl without further cleanup (PEG), and after precipitation by Ethanol-NaCl followed by cleanup using the Norgen Clean All kit (Nk)**. Two previously frozen samples from 3 cmbsf and 34 cmbsf at Aarhus Bay Station M1 were used as test material. We used two different DNA ladders, Lambda DNA/HindIII Marker (Thermo Scientific) to illustrate the large DNA fragment size spectrum, and GeneRuler 100 bp Plus DNA ladder (Fermentas) to illustrate the short DNA size spectrum.

Figure S8 **DNA yield comparisons of our extraction method using LP III to commercial DNA extraction kits by MO BIO (PowerSoil, PowerLyzer)**. All measurements done by fluorescence spectroscopy. **(A)** Aarhus Bay Station M1, 3.1 mbsf, and **(B)** Aarhus Bay Station M1, 10.5 mbsf. Bead-beating was used in extractions with the MOBIO PowerLyzer kit. Error bars indicate standard deviations of triplicate extractions.
