# Supplementary figures and images for: A modular method for the extraction of DNA and RNA, and the separation of DNA pools from diverse environmental sample types
Source: Front Microbiol. 2015 May 19;6:476. doi: 10.3389/fmicb.2015.00476 (PMC4436928; doi:10.3389/fmicb.2015.00476)

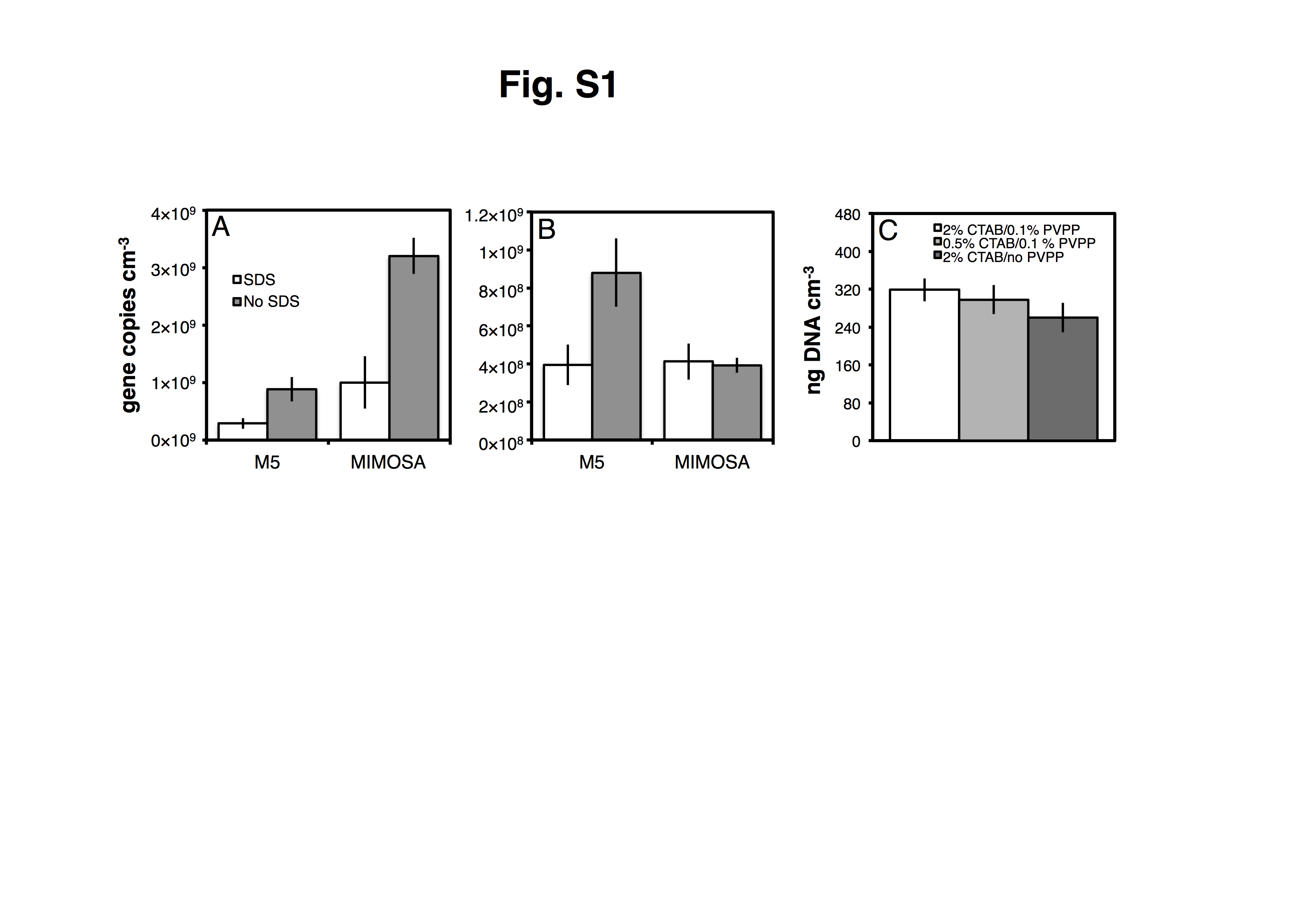

Supplement: Figure S1 — (A,B) 16S rRNA gene copy numbers cm−3 sediment corresponding to the DNA extracts shown in (Figure 2D (A: Bacteria; B: Archaea). (C) DNA yields obtained after incubating sediments from Aarhus Bay Station M5 with a second lysis solution containing two concentrations of CTAB (2%, 0.5%) and PVPP (0%, 0.1%). Error bars indicate standard deviations of triplicate DNA extractions. [file Image1.TIFF]

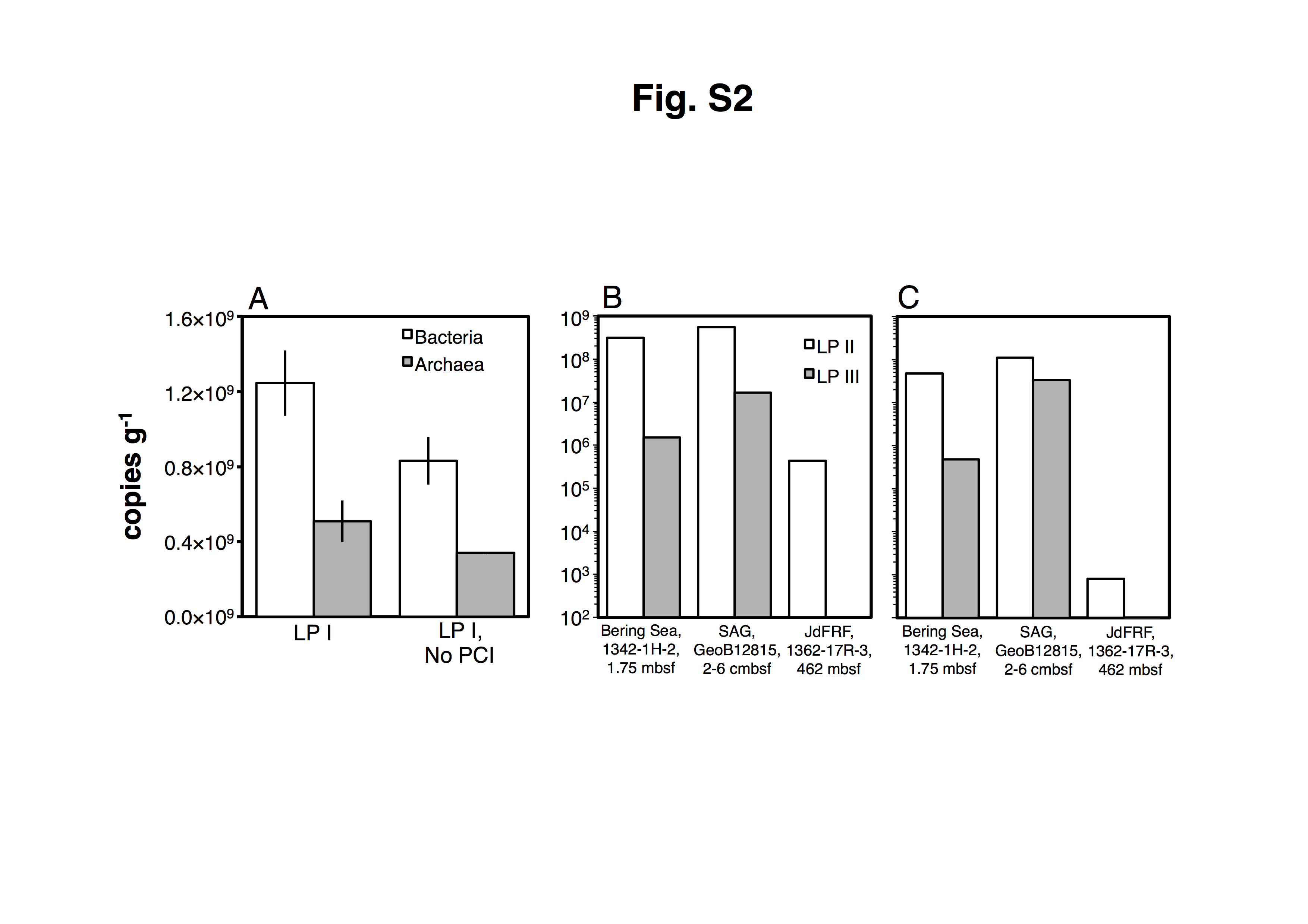

Supplement: Figure S2 — (A) Comparison of bacterial and archaeal 16S rRNA gene copy numbers after DNA extraction by LP I compared to LP I without PCI. Surface sediment from Aarhus Bay Station M1 was used as test material. Error bars indicate standard deviations of triplicate DNA extractions. (B,C) Comparison of bacterial (B) and archaeal (C) 16S rRNA gene copy numbers after DNA extraction by LP II compared to LP III from two oligotrophic sediments [Bering Sea, South Atlantic Gyre (SAG)] and subseafloor basalt (Juan de Fuca Ridge Flank). [file Image2.TIFF]

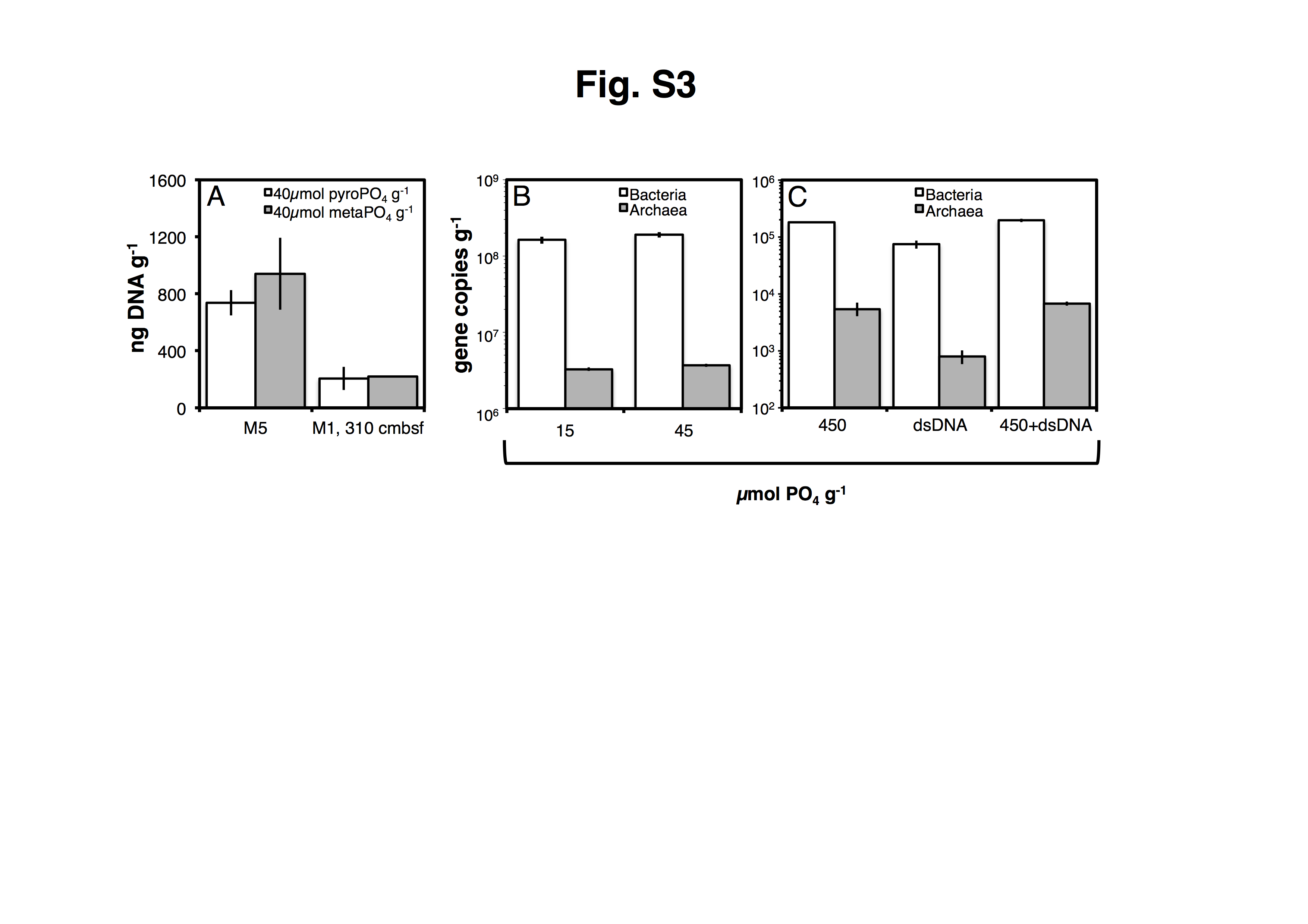

Supplement: Figure S3 — (A) Effect of adding the same concentration of pyrophosphate and hexametaphosphate on DNA yields from subsurface sediments of Aarhus Bay Station M5 and M1. Concentrations of pyrophosphate and hexametaphosphate correspond to 80 and 240 μmol PO4 g−1 sediment, respectively. (B) Bacterial and archaeal 16S rRNA gene copy numbers in DNA extracts from a Greenland glacial lake after two different amounts of PO4 had been added in the form of dNTPs (5 and 15 μmol dNTPs g−1). (C) Bacterial and archaeal 16S rRNA gene copy numbers in DNA extracts from Subglacial Lake Whillans. The PO4 treatments included 450 μmol PO4 g−1 as dNTPs (“450”), small amounts of dsDNA (“dsDNA”; 0.001 μmol PO4 g−1), and 450 μmol PO4 g−1 as dNTPs plus 0.001 μmol PO4 as dsDNA (“450+dsDNA”). Error bars indicate standard deviations of triplicate extractions (A), or ranges of duplicate extractions (B,C). [file Image3.TIFF]

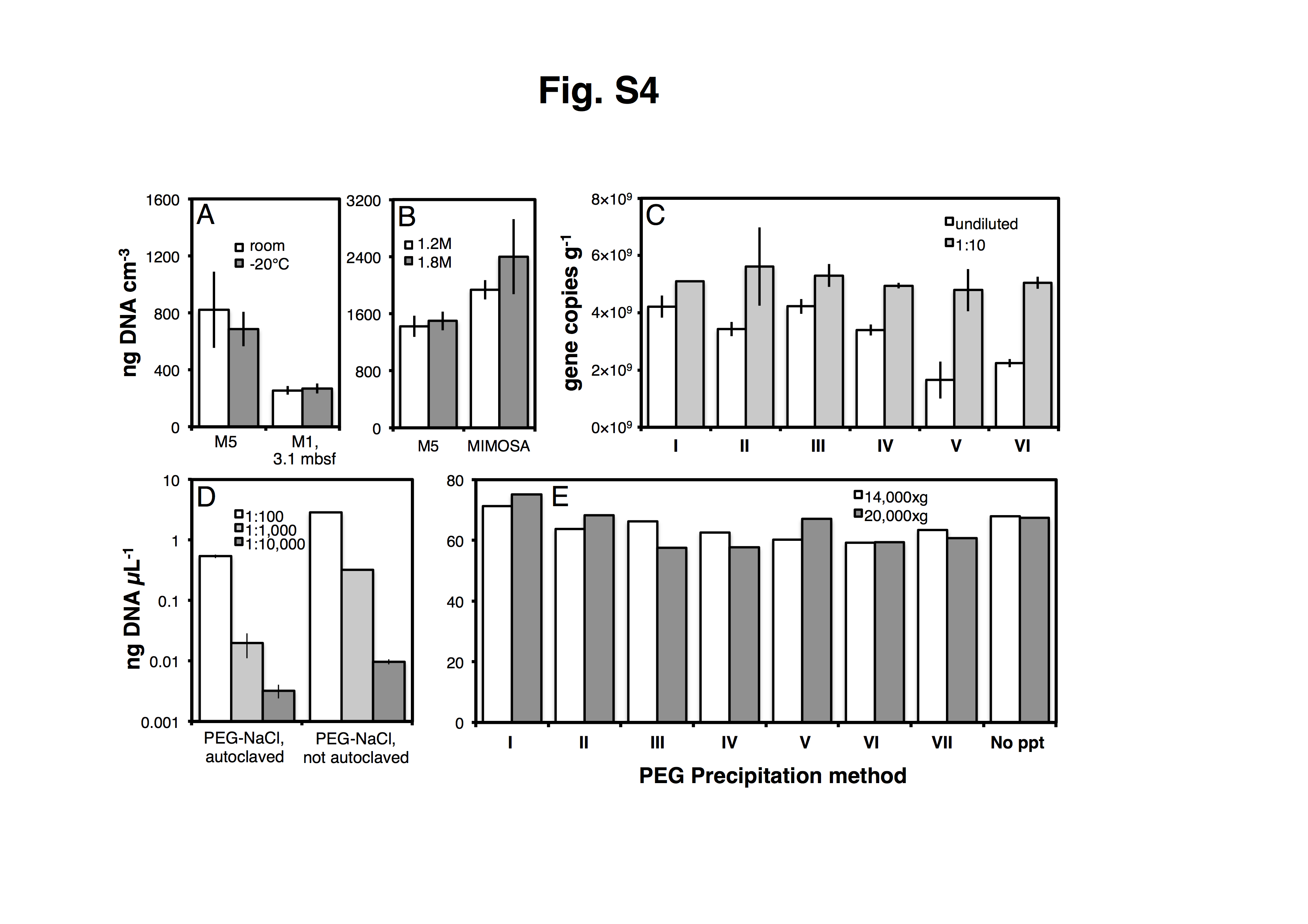

Supplement: Figure S4 — Comparison of DNA yields, expressed in ng DNA or gene copy numbers, using different precipitation methods. (A) DNA yields by Ethanol-NaCl precipitation at room temperature and at −20°C using DNA extracts from Aarhus Bay Stations M5 (1.2 mbsf) and M1 (3.1 cmbsf). (B) DNA yields by Ethanol-NaCl precipitation after LP III. The first treatment with ~1.2 M NaCl left from Lysis Solution II prior to ethanol addition, the second treatment with addition of 0.2 volumes of 5 M NaCl, which raised the NaCl concentration to ~1.8 M prior to ethanol addition. (C) Effect of precipitation method at two different dNTP additions on PCR inhibition based on quantifications of bacterial 16S rRNA gene copy numbers in original and tenfold diluted extracts. DNA was extracted from Aarhus Bay Station M1 surface sediment and PCR-amplified after precipitation, without further purification by the Norgen CleanAll kit. I–III: 15 μmol PO4 added g−1 sediment, IV–VI: 150 μmol PO4 g−1 sediment. Precipitation methods: I, IV: PEG-NaCl; II, V: PEG-ethanol-NaCl; III, VI: EtOH-NaCl. (D) Effect of autoclaving PEG-NaCl solution on DNA recovery from three dilutions of 100-bp ladder (original concentration: 500 ng DNA μL−1). (E) Effects of centrifugation force, MgCl2 addition, or manipulating the pH of the PEG 8000-NaCl solution with acetate buffers on DNA recovery by PEG 8000-NaCl precipitation. Aliquots of the same homogenized DNA extract from Aarhus Bay Station M5 were diluted tenfold and used for these tests. I: conventional PEG precipitation with 2 volumes of solution containing 30% PEG 6000-1.6 M NaCl (pH 8.3); II: as I, but amending PEG-NaCl solution with 0.1 volumes of 300 mM MgCl2 (pH 8.4); III: as I, but 0.1 volumes of 3 M Na acetate added to PEG solution (pH 8.7); IV: as I, but 0.1 volumes of 1:1 (v:v) 3 M acetic acid: 3 M Na acetate added (pH 4.6); V: as I, but 0.1 volumes of 9:1 (v:v) 3 M acetic acid: 3 M Na acetate added (pH 3.7); VI: as I, but 0.1 volumes of 99:1 (v:v) 3 M acetic acid: 3 M Na ace [file Image4.TIFF]

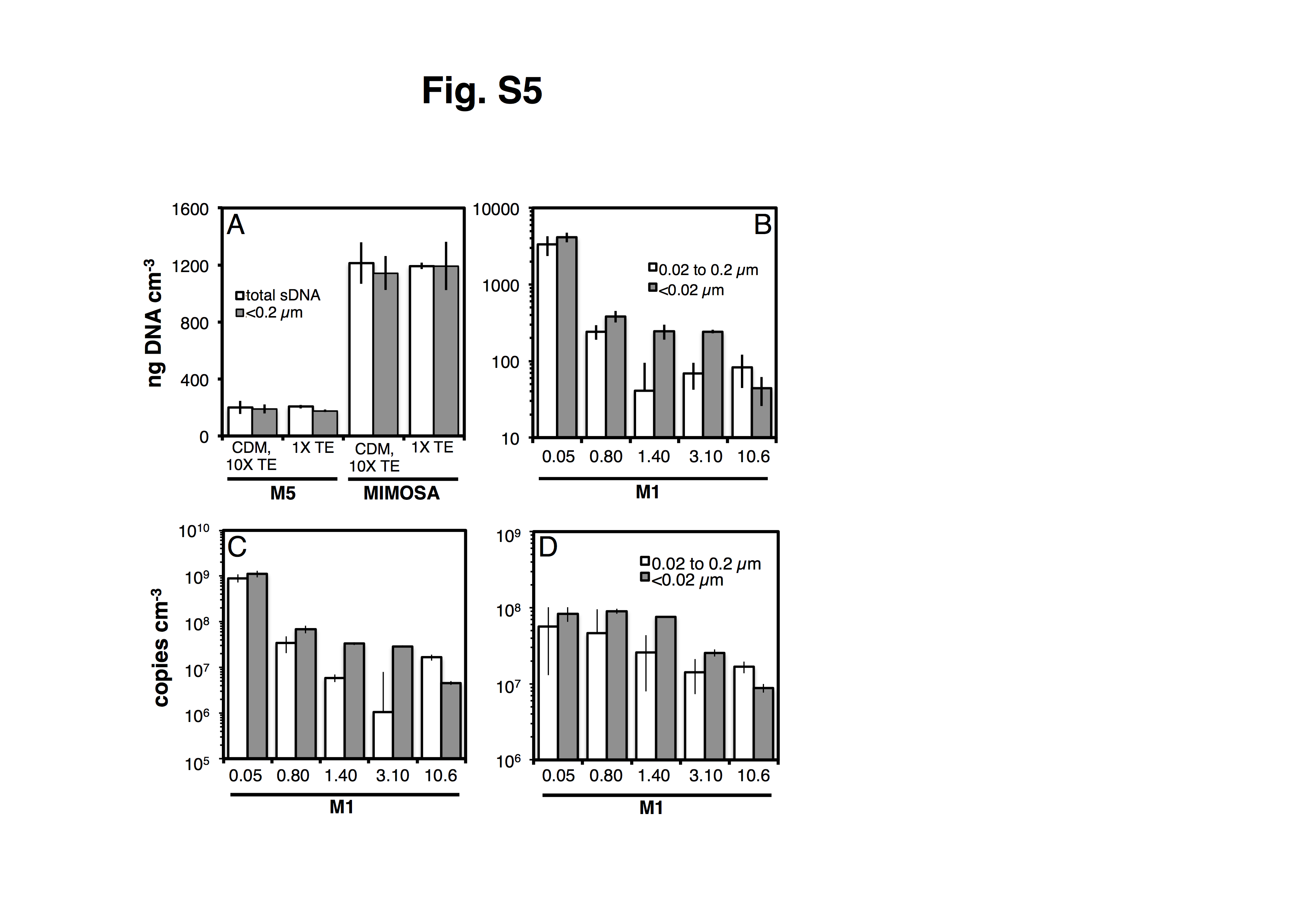

Supplement: Figure S5 — (A) Amount of sDNA that passes through a 0.2-μm filter compared to total sDNA. Samples were from Aarhus Bay Stations M5 and MIMOSA. sDNA was extracted by the same method as in (Figure 8C. (B) Amount of sDNA in the 0.02–0.2 μm fraction compared to the amount of sDNA in the <0.02 μm fraction. Data from five different depths at Aarhus Bay Station M1, of which the uppermost depths (0.05 mbsf, 0.80 mbsf, 1.40 mbsf, 3.10 mbsf) correspond to marine sediment, and the lowermost depth (10.5 mbsf) corresponds to a soil layer. (C,D) Bacterial (C) and archaeal (D) qPCR results on the same DNA fractions and samples as shown in (B). Error bars indicate standard deviations of triplicate extractions. [file Image5.TIFF]

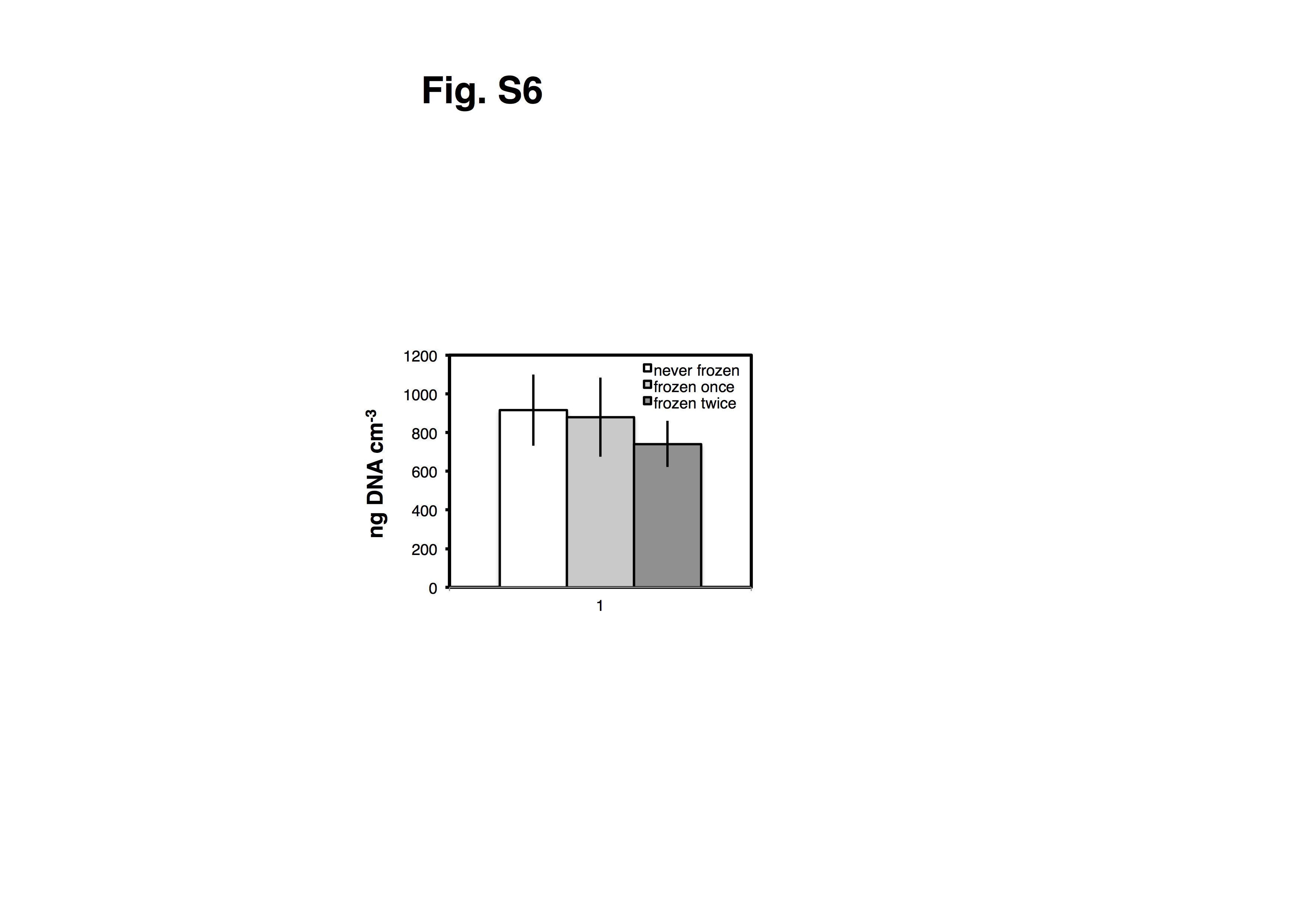

Supplement: Figure S6 — Effect of freezing on nsDNA yields from Aarhus Bay Station M5. Error bars indicate standard deviations of triplicate extractions. [file Image6.TIFF]

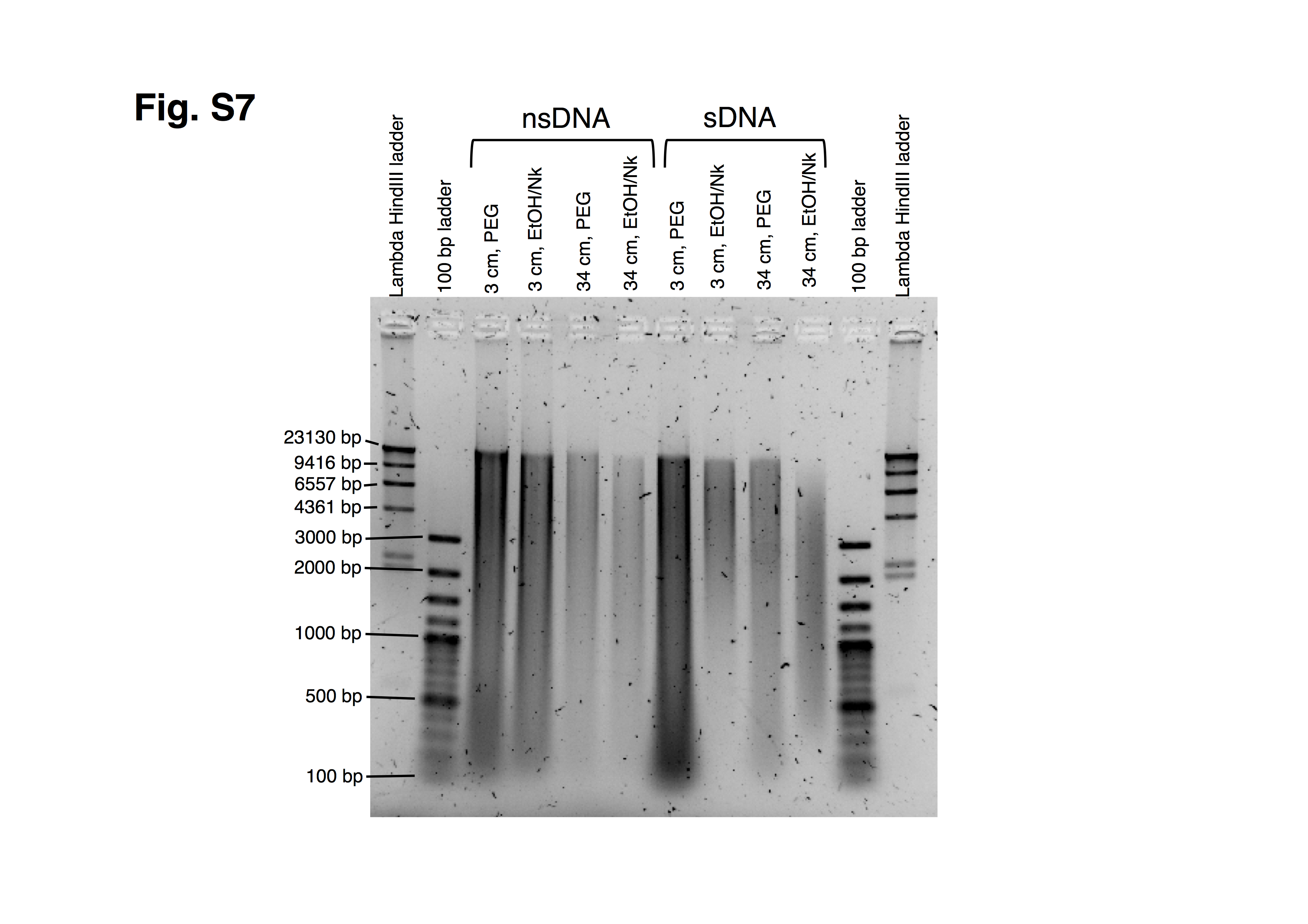

Supplement: Figure S7 — DNA size distributions after nsDNA and sDNA extract precipitation by PEG 8000-NaCl without further cleanup (PEG), and after precipitation by Ethanol-NaCl followed by cleanup using the Norgen Clean All kit (Nk). Two previously frozen samples from 3 cmbsf and 34 cmbsf at Aarhus Bay Station M1 were used as test material. We used two different DNA ladders, Lambda DNA/HindIII Marker (Thermo Scientific) to illustrate the large DNA fragment size spectrum, and GeneRuler 100 bp Plus DNA ladder (Fermentas) to illustrate the short DNA size spectrum. [file Image7.TIFF]

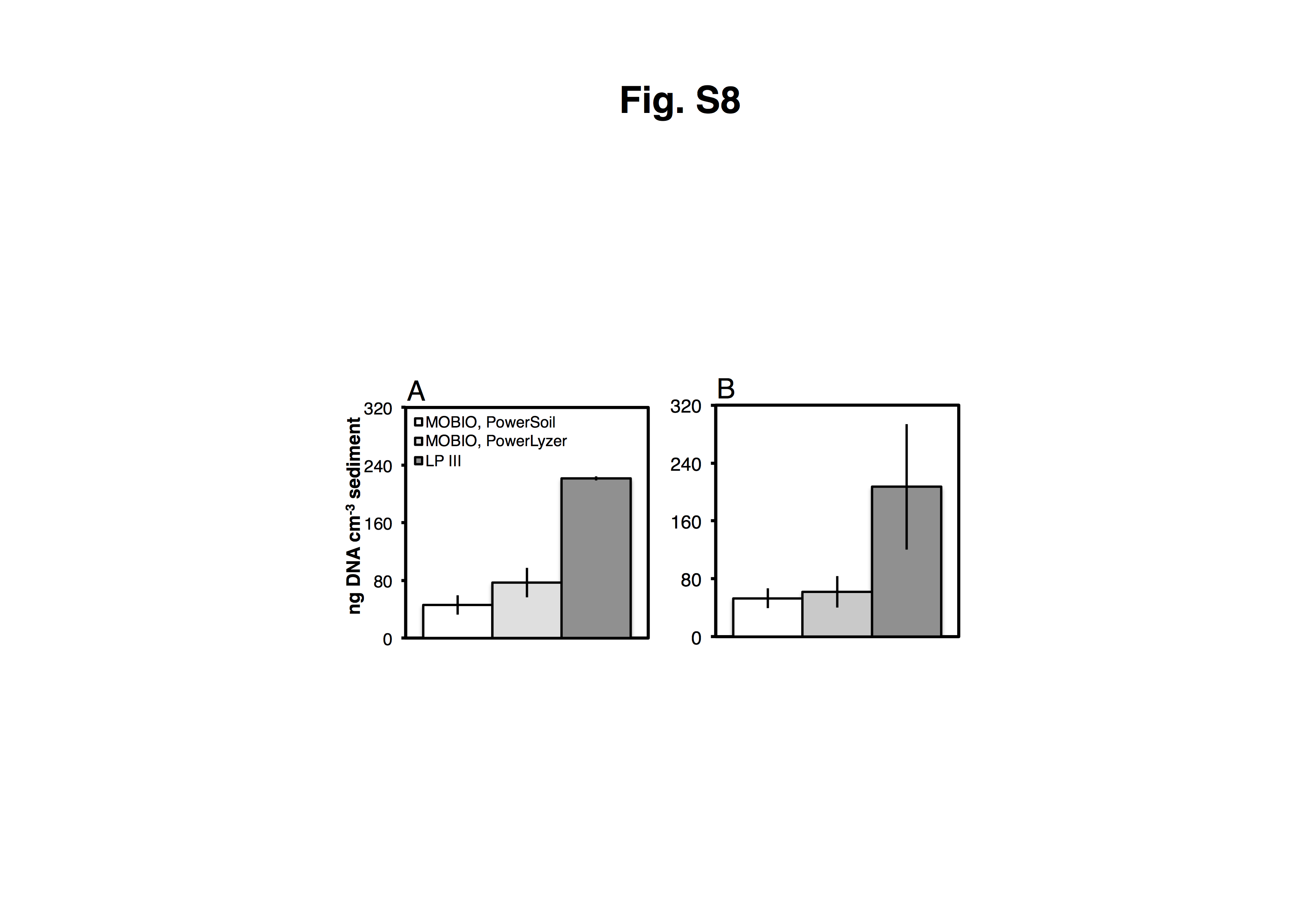

Supplement: Figure S8 — DNA yield comparisons of our extraction method using LP III to commercial DNA extraction kits by MO BIO (PowerSoil, PowerLyzer). All measurements done by fluorescence spectroscopy. (A) Aarhus Bay Station M1, 3.1 mbsf, and (B) Aarhus Bay Station M1, 10.5 mbsf. Bead-beating was used in extractions with the MOBIO PowerLyzer kit. Error bars indicate standard deviations of triplicate extractions. [file Image8.TIFF]
