# Supplementary material for: A modular method for the extraction of DNA and RNA, and the separation of DNA pools from diverse environmental sample types
Source: Front Microbiol. 2015 May 19;6:476. doi: 10.3389/fmicb.2015.00476 (PMC4436928; doi:10.3389/fmicb.2015.00476)
Supplement: Supplementary file 9 [file DataSheet1.DOCX]

**Supplementary text**

**Nucleic acid extraction from filters**

Four different extraction protocols were compared: RNA PowerSoil® Total RNA Isolation Kit adapted to the Sterivex^TM^ filter units, PowerWater® Sterivex™ DNA Isolation Kit adapted for RNA extraction (PowerWater® Sterivex™ RNA Protocol, MO BIO Laboratories), and two permutations of our protocol.

As the names indicate, the two commercial kits are designed for extractions from water and soil, respectively. The PowerWater® Sterivex™ Isolation Kit is designed to isolate nucleic acids directly from the Sterivex™ filter units, and we followed the manufacturer’s protocol for the extraction of RNA. The RNA PowerSoil® Total RNA Isolation Kit was adapted for the extraction from filter units by adding Bead Solution, Solution SR1 and Solution SR2 directly to the Sterivex^TM^ filter units and then following steps 4-6 and 13-15 of the PowerWater® Sterivex™ RNA Protocol.

Our protocol involved initially adding 0.1 ml of 10 mM dNTP solution and 0.5 mL of Lysis Solution I to the Sterivex^TM^ filter units. The filter units were then capped and vortexed at maximum speed on a Vortex Genie for 10 minutes. To ensure complete exposure of filters to dNTPs and lysis solution I, the filters were rotated by 180°C after 5 min. One protocol permutation then included a freeze-thaw+heat incubation step, where filters were frozen at –80°C and then incubated for one hour on a Thermomixer Comfort (Eppendorf) at 50°C and 600 rpm. The solution containing nucleic acids was aspirated using a syringe and transferred to a Falcon tube. Nucleic acid extracts were washed with chloroform-isoamylalcohol, precipitated with isopropanol-NaCl, and purified using the CleanAll RNA/DNA Clean-up and Concentration Kit (Norgen Biotek).

**Whole-cell extraction and quantification**

To test whether sediment handling and sDNA extractions resulted in significant cell lysis, we counted SYBR-Green stained cells in the sediment pellet obtained after the final centrifugation step. After extracting sDNA from Aarhus Bay Station M5 according to the protocols of Ogram *et al.* (1987), Corinaldesi *et al.* (2005), and this protocol, the remaining sediment pellet after the final centrifugation step was fixed with 1.8 mL of 3% (*wt/vol*) NaCl and 2% (*vol/vol*) formaldehyde.

After incubation overnight at 4°C, samples were prepared for direct cell counts according to the following procedure, which was adapted from published protocols by Kallmeyer *et al.* (2008) and Langerhuus *et al.* (2012). Sediment slurries were tenfold diluted using 3% NaCl solution. One hundred μL of diluted slurry were mixed with 600 μL 3% NaCl solution, 100 μL detergent solution (3% NaCl, 100 mM Na_2_EDTA, 100 mM sodium pyrophosphate, 1% vol/vol Tween-80), and 100 μL of methanol, and shaken at 1200 rpm for 1 hour at room temperature on a Thermomixer (Eppendorf). Samples were centrifuged at 100×*g* for 30 seconds, and supernatants, containing suspended cells, transferred to clean tubes on ice. The sediment pellets were treated again with 600 μl of 3% NaCl solution, 100 μL of detergent solution and 100 μL of methanol, and sonicated for 1 minute on ice with a Sonopuls Homogenizer HD2070 (Bandelin) set to 20% power output. Samples were shaken once more at 1200 rpm for 1 hour at room temperature and centrifuged at 100×*g* for 30 seconds. The resulting supernatants were merged with the first supernatants, while the pellets were mixed with 800 μL of 3% NaCl solution, vigorously vortexed for 10 seconds, and the resulting suspension was also combined with the first supernatant. The combined cell extracts were then run through a 40-μm nylon mesh (Cell Stainer 40 μm Nylon, BD Falcon), followed by a wash with 5 mL of 3% NaCl, and filtered onto black polycarbonate filters (25-mm diameter, 0.22-μm pore size, Millipore GTBP02500). Potential remaining carbonate particles and/or precipitates were eliminated by applying 2 ml of a solution containing 0.1 N HCl and 3% NaCl directly onto the filter for 5 minutes. Filters were washed with 5 mL TE buffer (10 mM Tris-Cl, 1 mM EDTA, 3% NaCl, pH 8.0), air-dried and cut in quarters. Cells on filters were stained with SYBR-Green I and counted on an automated microscope system as described in Morono *et al.* (2009). The described procedure was also carried out on samples that did not undergo any sDNA extraction to check the initial number of cells in the sediment, and on controls without sediment to check for contamination.

**Recipes for Extraction Reagents**

*Carbonate Dissolution Mix (CDM)*

430 mM sodium acetate (35.3 g L^-1^)

430 mM glacial acetic acid (27.1 mL L^-1^)

10mM sodium hexametaphosphate (6.1 g L^-1^)

3% sodium chloride (30 g L^-1^)

*10× TE Buffer, pH 10*

300 mM tris hydrochloride (36.3 g L^-1^)

10 mM EDTA (3.4 g L^-1^)

3% sodium chloride (30 g L^-1^)

Adjust to pH 10 with NaOH.

*1× TE Buffer, pH 10*

Dilute 10x TE Buffer tenfold with dH_2_O.

*Lysis solution I, pH 10*

30 mM tris hydrochloride (3.63 g L^-1^)

30 mM EDTA (10.2 g L^-1^)

800 mM guanidine hydrochloride (76.4 g L^-1^)

0.5% Triton X-100 (5 mL L^-1^)

Adjust to pH 10 with NaOH.

*Cell Lysis Solution II*

2% CTAB (20 g L^-1^)

0.1% PVPP (1 g L^-1^)

2.5M sodium chloride (146 g L^-1^)

**References**

Corinaldesi, C., Danovaro, R., Dell’Anno, A. (2005). Simultaneous recovery of extracellular and

intracellular DNA suitable for molecular studies from marine sediments. *Appl. Environ.*

*Microbiol.* 71, 46-50.

Kallmeyer, J., Smith, D. C., Spivack, A. J., D’Hondt, S. (2008). New cell extraction procedure

applied to deep subsurface sediments. *Limnol. Oceanogr. Meth.* 6, 236-245.

Langerhuus AT, Røy H, **Lever MA**, Morono Y, Inagaki F, Jørgensen BB, Lomstein BA (2012)

Endospore abundance and D:L amino acid modeling of bacterial turnover in Holocene marine

sediment (Aarhus Bay). *Geochim. Cosmochim. Acta* 99, 87-99.

Morono, Y., Terada, T., Masui, N., Inagaki, F. (2009). Discriminative detection and enumeration of

microbial life in marine subsurface sediments. *ISME J*. 3, 503-511.

Ogram, A., Sayler, G. S., Barkay, T. (1987). The extraction and purification of microbial DNA from

sediments. *J. Microbiol. Meth.* 7, 57-66.
